# Genomic analysis uncovers functional variation in the C-terminus of anthocyanin-activating MYB transcription factors

**DOI:** 10.1038/s41438-021-00514-1

**Published:** 2021-04-01

**Authors:** Jessica A. Rodrigues, Richard V. Espley, Andrew C. Allan

**Affiliations:** 1 The New Zealand Institute for Plant and Food Research Limited, 120 Mount Albert Road, Sandringham, Auckland, 1025 New Zealand; 2 School of Biological Sciences, University of Auckland, 3A Symonds St, Auckland, 1010 New Zealand

**Keywords:** Plant molecular biology, Transcriptional regulatory elements

## Abstract

MYB transcription factors regulate diverse aspects of plant development and secondary metabolism, often by partnering in transcriptional regulatory complexes. Here, we harness genomic resources to identify novel MYBs, thereby producing an updated eudicot MYB phylogeny with revised relationships among subgroups as well as new information on sequence variation in the disordered C-terminus of anthocyanin-activating MYBs. BLAST® and hidden Markov model scans of gene annotations identified a total of 714 MYB transcription factors across the genomes of four crops that span the eudicots: apple, grape, kiwifruit and tomato. Codon model-based phylogenetic inference identified novel members of previously defined subgroups, and the function of specific anthocyanin-activating subgroup 6 members was assayed transiently in tobacco leaves. Sequence conservation within subgroup 6 highlighted one previously described and two novel short linear motifs in the disordered C-terminal region. The novel motifs have a mix of hydrophobic and acidic residues and are predicted to be relatively ordered compared with flanking protein sequences. Comparison of motifs with the Eukaryotic Linear Motif database suggests roles in protein–protein interaction. Engineering of motifs and their flanking regions from strong anthocyanin activators into weak activators, and vice versa, affected function. We conclude that, although the MYB C-terminal sequence diverges greatly even within MYB clades, variation within the C-terminus at and near relatively ordered regions offers opportunities for exploring MYB function and developing superior alleles for plant breeding.

## Introduction

MYB transcription factors (TFs) regulate diverse aspects of metabolism and development across eukaryotes^1^. In plants, the MYB family has undergone relatively large expansions during the transition to life on land and the development of the seed habit^2^. Seed-bearing plants possess hundreds of MYB TFs^3^, with different MYBs regulating diverse pathways such as stress responses, hormone signalling, organ development, reproduction, and secondary metabolite production^4,5^. Several fruit and vegetable crop traits are MYB-regulated, including colour, flavour, texture, nutritional content, and storage life^4^. Given the many adaptive and reproductive plant traits which MYBs regulate, MYB family expansion was likely an underlying factor in the complex assortment of cell fates plants evolved in order to thrive as sessile land organisms^3,5^.

The MYB TF family is defined by one or more characteristic conserved MYB repeats in the DNA-binding domain, each of which consists of a helix-turn-helix motif^6^. Most plant MYB proteins contain two tandem N-terminal MYB repeats that are similar to the second and third domains of vertebrate MYBs. Thus, MYBs of this class are referred to as 2R or R2R3 MYBs. Other plant MYB family members contain either just a single R3 MYB repeat (R3 MYBs), three MYB repeats (R1R2R3 or 3R MYBs, the most common vertebrate class), or four MYB repeats (R1R2R2R1/2 or 4R MYBs)^7^. In contrast to the well-structured and conserved DNA-binding domains of the N-terminus, the larger C-terminal regions of R2R3 MYBs appear to be principally unstructured, varying greatly in length and sequence^8^. Such extended regions of intrinsic disorder are enriched among eukaryotic TFs compared with other proteins, associated with the increased presence of molecular recognition features (MoRFs) that participate in dynamic protein–protein interactions^9^. MoRFs are often identified as short linear motifs (SLiMs) of 3 to 11 amino acids that are evolutionarily variable, yet contain a few relatively conserved specificity-determining residues favouring structural order within a larger disordered context^10–13^. Interactions at SLiMs tend to be specific yet low affinity, thus ideal for regulation, signalling and post-translational modification^10,14^. It is widely thought that the loose constraints on protein structure and sequence at MoRFs have facilitated the evolution of TF regulation by varied cellular signalling pathways and interacting co-factors, thereby contributing to eukaryotic diversity and complexity^9,10,12–14^. Hence, although the C-termini of plant MYBs are poorly conserved, they probably confer functional specialisation and adaptive regulation in a family where members vary greatly in their roles^15^.

Previous studies have categorised MYBs into over 25 subgroups based on phylogeny and function^3,7^, with several subgroups displaying SLiM-like C-terminal sequence motifs that are conserved among their members^16^. Some of these conserved C-terminal sequence motifs are verified interaction sites, such as that with co-activating basic helix–loop–helix (bHLH) TFs at the subgroup 12 motif^8^ and that with repressive chromatin modifiers at one of the subgroup 4 motifs^17^. It is noteworthy that, although MYBs of subgroups 2, 5, 6, 15, 16, and 19 also require a bHLH partner to activate target promoters, the bHLH-interacting site for subgroups 5, 6, 15, and 16 occurs within the N-terminal R3 MYB sequence^5,8,18–20^. Thus, bHLH interaction of subgroup 12 MYBs appears to have evolved convergently with that of subgroups 5, 6, 15, and 16, illustrating the power of SLiMs in ex nihilo evolution of protein regulation^8,14^. Indeed, most SLiM classes include examples of convergent evolution^11^. In addition to co-activator requirements, MYB function is regulated at transcriptional, post-transcriptional and post-translational levels^4,7,21–24^. The C-terminal motif previously described for subgroup 6^16^, which includes anthocyanin-activating MYBs, coincides with the target sequence of trans-acting small interfering RNA TAS4-siRNA81(-)^25^. It is likely that some conserved C-terminal motifs also contribute to post-translational regulation, serving either as docking or target sites for modification machinery^12^.

Here, we explore MYB diversity and its functional implications, to enhance resources for crop improvement. We examine diversity and molecular evolution with the latest evolutionary models and resources for five mature reference genomes that span eudicots: *Arabidopsis thaliana*, *Malus* × *domestica* (apple), *Vitis vinifera* (grape), *Actinidia chinensis* (kiwifruit), and *Solanum lycopersicum* (tomato). We identify several new MYB family members in apple and kiwifruit while providing firmer support for some previously ambiguous phylogenetic relationships of MYB subgroups. Finally, by characterising novel members of the horticulturally important clade of anthocyanin-activating MYBs, we demonstrate how novel putative MoRFs in the disordered C-terminus enhance anthocyanin activation in functional assay.

## Results

### Enhanced annotation of MYB genes

**Fig. 1:**
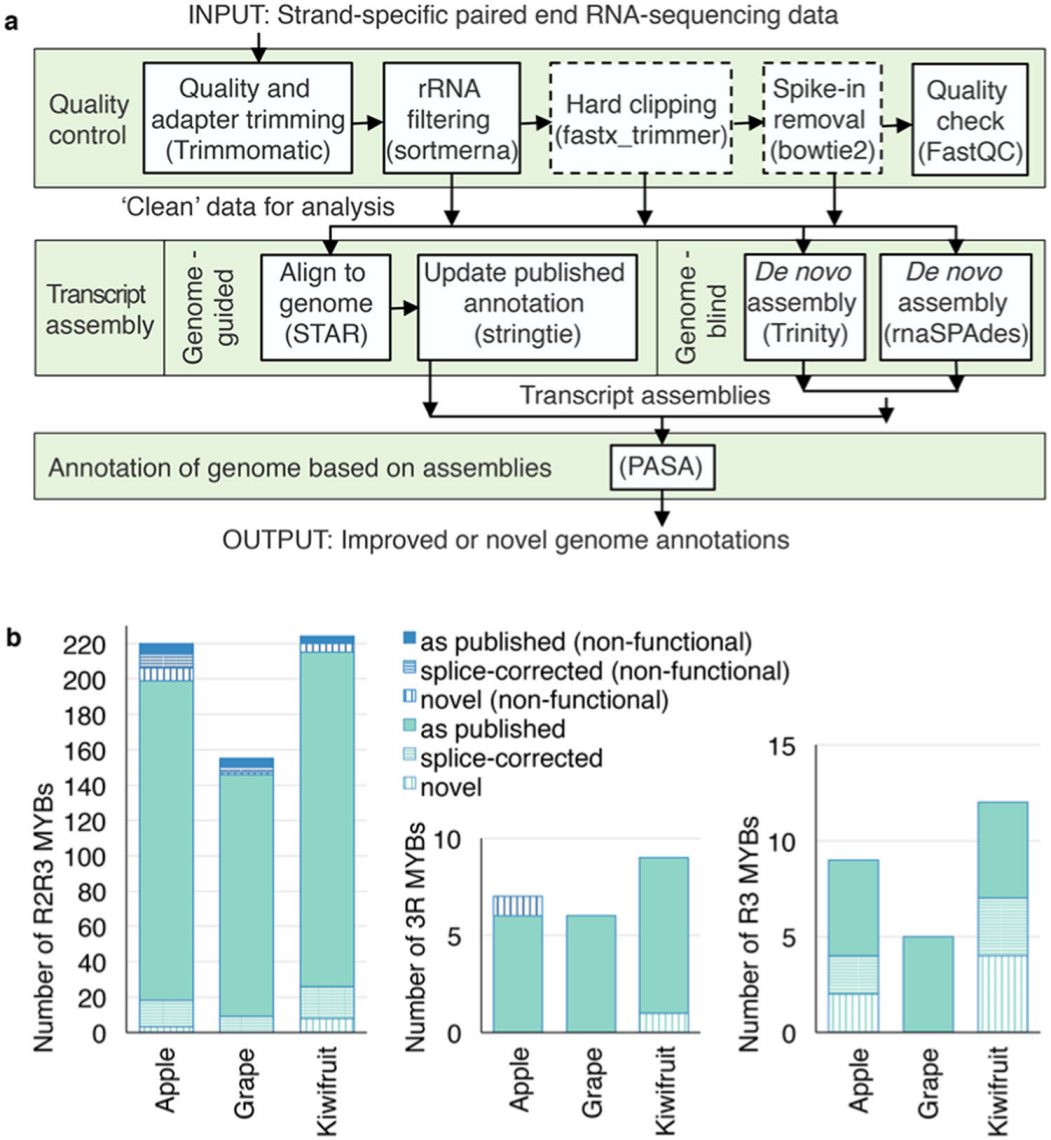
High quality RNA-sequencing data enhanced genome-wide detection of MYB genes and improved the annotation of dominant splice isoforms. **a** Bioinformatic pipeline to obtain improved and novel gene annotations of published reference genomes using RNA-sequencing data. Publicly available tools used are indicated in brackets; dashed boxes indicate steps applied to some but not all datasets. **b** Numbers of R3, R2R3 and 3R MYBs identified in apple, kiwifruit and grape reference genomes, including novel genes not in published genome annotations and ones for which the dominant splice sites were corrected using RNA-sequencing data. Genes that contain frameshift mutations or premature termination codons in the MYB domain are indicated as non-functional

Since *Arabidopsis thaliana* and *Solanum lycopersicum* (tomato) genomes have had extensive collaborative input in assembly and gene annotation^26,27^, we performed hidden Markov model and BLAST^®^ searches on the latest gene annotations, i.e. TAIR10 for *Arabidopsis* and ITAG4.0 for tomato. Our approach did not identify any *Arabidopsis* MYB genes beyond those already described^7^ but found four new genes for tomato^28^. For the newer apple, kiwifruit and grape genomes, we first enhanced existing gene annotations by harnessing in-house and publicly available RNA sequencing (RNA-seq) data that spanned tissues and cultivars. Subsequent hidden Markov model and BLAST search identified MYBs spanning R3, R2R3 and 3R classes (Fig. 1a, b; Dataset S3). Our analyses excluded a minor subset of genes (~10%) that produce non-functional proteins from the reference genome sequence based on frameshifts or deletions of the N-terminal DNA binding domain. Thus, we finally identified 215 functional R2R3 MYBs in the kiwifruit genome compared with 155^29^ and 93^30^ from previous studies that used either the same reference genome or an earlier version from a different cultivar, respectively. We identified 199 functional apple R2R3 proteins, of which 23 were absent from the lists of 186^31^ and 222^32^ R2R3 MYBs generated using an earlier version of the apple genome^33^. Unresolved heterozygosity resulted in the unintentional inclusion of both haplotypes in the sequence of this earlier assembly, and thus alleles were erroneously identified as distinct genes^34^. We did not identify functional grape MYBs beyond those previously described^35^.

RNA-seq data corrected the dominant splice isoform annotation for 64 MYB genes across apple, kiwifruit and grape genomes and identified a further 33 MYB genes that were not previously annotated, with three apple genes and one kiwifruit gene failing to map to the reference genome (Fig. 1b). The overall improvement to existing annotations based on our approach was assessed by comparing de novo transcripts over 1 kb long, the revised gene annotations derived from them and the original published gene annotations with which they overlap (Fig. S1). On average, de novo transcripts and the revised annotations derived from them produced similar results in terms of transcript length, protein length and mapping of RNA-seq reads, to outperform the original published annotations for apple and kiwifruit and a significant improvement for grape. This could be attributed to enhanced definition of splice sites and untranslated regions.

Our approach of validating gene annotations through mapping of de novo assembled transcripts rather than mapping of unassembled reads reduced mis-mapping at recently duplicated genes. For example, *Vitvi02g01019* (*MYBA1*) is one of 12 tandemly duplicated grape *MYBA* genes clustered on chromosome 2. Although unassembled reads map to *MYBA1* in the Sauvignon blanc dataset, de novo assembled transcripts from the dataset map only to *MYBA3* (*Vitvi02g01024*) and *MYBA2* (*Vitvi02g01015*). A search for *VvMYBA1*-specific single nucleotide polymorphisms (SNPs) across datasets from different grape varieties found that only one (Nebbiolo) de novo transcript possessed signature SNPs within the coding sequence; however, the rest of the transcript displayed characteristic *VvMYBA2* and *VvMYBA3* SNPs. Thus, there was no significant support for the expression of *VvMYBA1* in any of the datasets we analysed, but there was support for the expression of *VvMYBA3* in Sauvignon blanc, Pinot noir and Nebbiolo and *VvMYBA2* in Sauvignon blanc, Pinot noir, Tempranillo and Nebbiolo datasets.

### Improved support for clading within and among subgroups

**Fig. 2:**
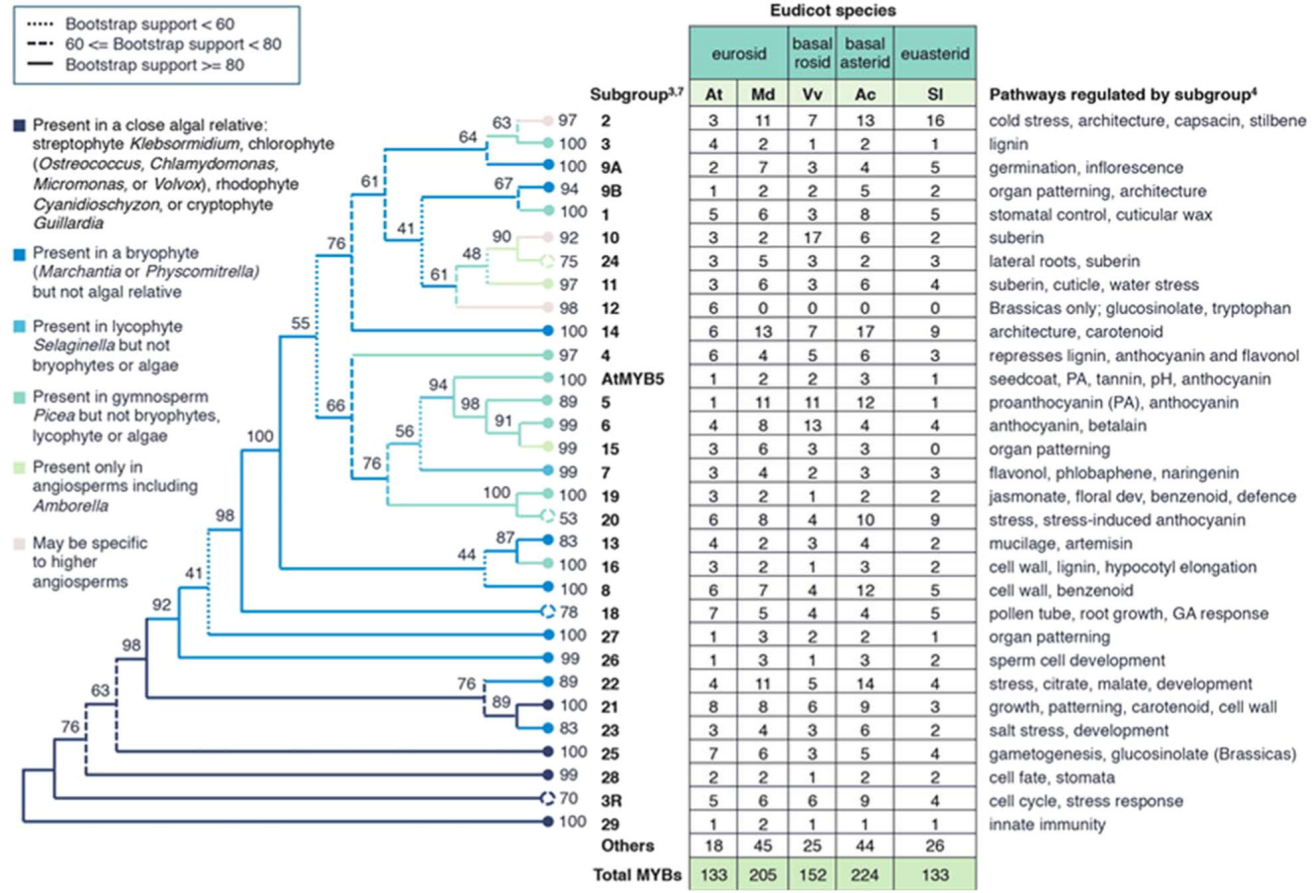
Phylogenetic summary of functional R2R3 and 3R MYBs identified in *Arabidopsis*, apple, grape, kiwifruit and tomato genomes. Numbers on the maximum likelihood phylogenetic tree indicate support based on 10,000 ultrafast bootstrap replicates, while numbers in the table indicate the number of subgroup members in each species. R2R3 subgroup nomenclature, function, and presence in early diverging plants is based on previous publications ^3,4,7,50^. MYBs across the phylogeny that do not fall into the subgroups originally described^7^ are placed in the “Others” category. Reference genomes and/or transcriptomes used are: At *Arabidopsis thaliana* TAIR10, Sl *Solanum lycopersicum* (tomato) ITAG4.0, Md *Malus* x *domestica* (apple) GDDH13 v1.1, Vv *Vitis vinifera* 12x v3 with the Vcost.V3 annotation, Ac *Actinidia chinensis* (kiwifruit) Red5

Our process of applying the same MYB annotation pipeline to multiple species allowed direct comparison of subgroup membership across species (Fig. 2). Variation in total numbers of MYBs among species reflects relatively recent genome duplications in apple and kiwifruit compared with grape, tomato and *Arabidopsis*. Both the maximum likelihood phylogenetic tree built with iqtree using a codon alignment of functional R2R3 and 3R MYBs (*Arabidopsis*, apple, grape, kiwifruit and tomato; Figs. 2, S2), as well as that built with RAxML using an amino acid alignment of functional and non-functional R3, R2R3 and 3R MYBs (*Arabidopsis*, apple, grape and kiwifruit; Fig. S3), indicate with moderate to high support (bootstrap of 92 in Fig. S2 and 79 in Fig. S3) that all land plant-specific eudicot R2R3 subgroups except subgroups 22 and 23 belong to a single land plant-specific clade. Based on the functions of the early diverging subgroups within this large clade, it is likely that its ancestral algal R2R3 MYB played a role in growth and development before the transition to life on land. Both phylogenetic trees also converge in placing the clade of subgroups 21, 22 and 23 as sister to the large land plant-specific clade (bootstrap of 98 in Fig. S2 and 75 in Fig. S3). Current roles for these subgroups also indicate an ancestral role in growth and development. Thus, all land plant-specific eudicot R2R3 MYB subgroups owe their origin to only two of at least five ancient algal R2R3 MYB clades. There appears to have been stricter selection against expansion and diversification in the ancient 3R MYB subgroup and R2R3 subgroups 29 (CDC5-like), 28 (AtMYB88, AtMYB124) and 25. This is likely due to lower tolerance of perturbations in the pathways they regulate: cell cycle, innate immunity, cell fate and gametogenesis, respectively^4,7^.

The two trees also concur in defining membership within R2R3 MYB subgroups, confirming the monophyly of all subgroups except 18, 20, and 24. However, the two trees do differ in support and topology of relationships among different subgroups. Codon evolutionary models generally outperform amino acid models^36,37^; nonfunctional MYB genes are more likely to possess homoplasies, and sampling of fewer species is more likely to result in failure to detect homoplasies, resulting in an inferior phylogeny^38^. In line with this, inter-subgroup relationships are more strongly supported in the tree built using only functional R2R3 and 3R MYBs from *Arabidopsis*, apple, grape, kiwifruit and tomato (Fig. S2) compared with the other constructed tree (Fig. S3).

Within the clade of land plant-specific subgroups, the early divergence of development-related subgroups 18, 26 and 27 results in a large highly supported clade (bootstrap of 100) of subgroups 1 to 17, 19, 20 and 24, where regulation of secondary metabolism is the predominant function. MYB family expansion during the evolution of the seed habit is due to the expansion of this clade of secondary metabolism regulators. The highly supported clade (bootstrap of 94) of anthocyanin- and proanthocyanin-regulating subgroups AtMYB5, 5 and 6 evolved during that large expansion, probably from a flavonoid regulator based on the flavonol- and flavone-regulating functions of its sister, subgroup 7. These form a moderately supported (bootstrap of 76) clade together with subgroups 19 and 20, which mostly play roles in development and response to biotic and abiotic stress. The placement of angiosperm-specific organ patterning-related subgroup 15 as the closest sister to subgroup 6 is well-supported (bootstrap of 91).

### Early diverging subgroup 6 members do not activate anthocyanin

Subsequent analysis focused on MYB subgroup 6, whose membership was identical in both phylogenetic trees and included previously undescribed members. For all species except grape, the closest homologs of described anthocyanin-promoting members were other members from the same species rather than putative orthologs from other species. Analysis could not differentiate whether this resulted from gene conversion among subgroup 6 members of the same species, restrictions placed by co-evolution with binding partners or independent replication from a single ancestral eudicot anthocyanin regulator in all five species. Grape anthocyanin activators fall into a clade of 12 tandem replicates located on chromosome 2 and a second clade of three tandem replicates on chromosome 14. The chromosome 2 clade includes genes that mostly contribute to fruit colour, such as the functionally redundant *VvMYBA1* and *VvMYBA2*^39^, while the chromosome 14 clade appears to colour vegetative tissues^40^.

**Fig. 3:**
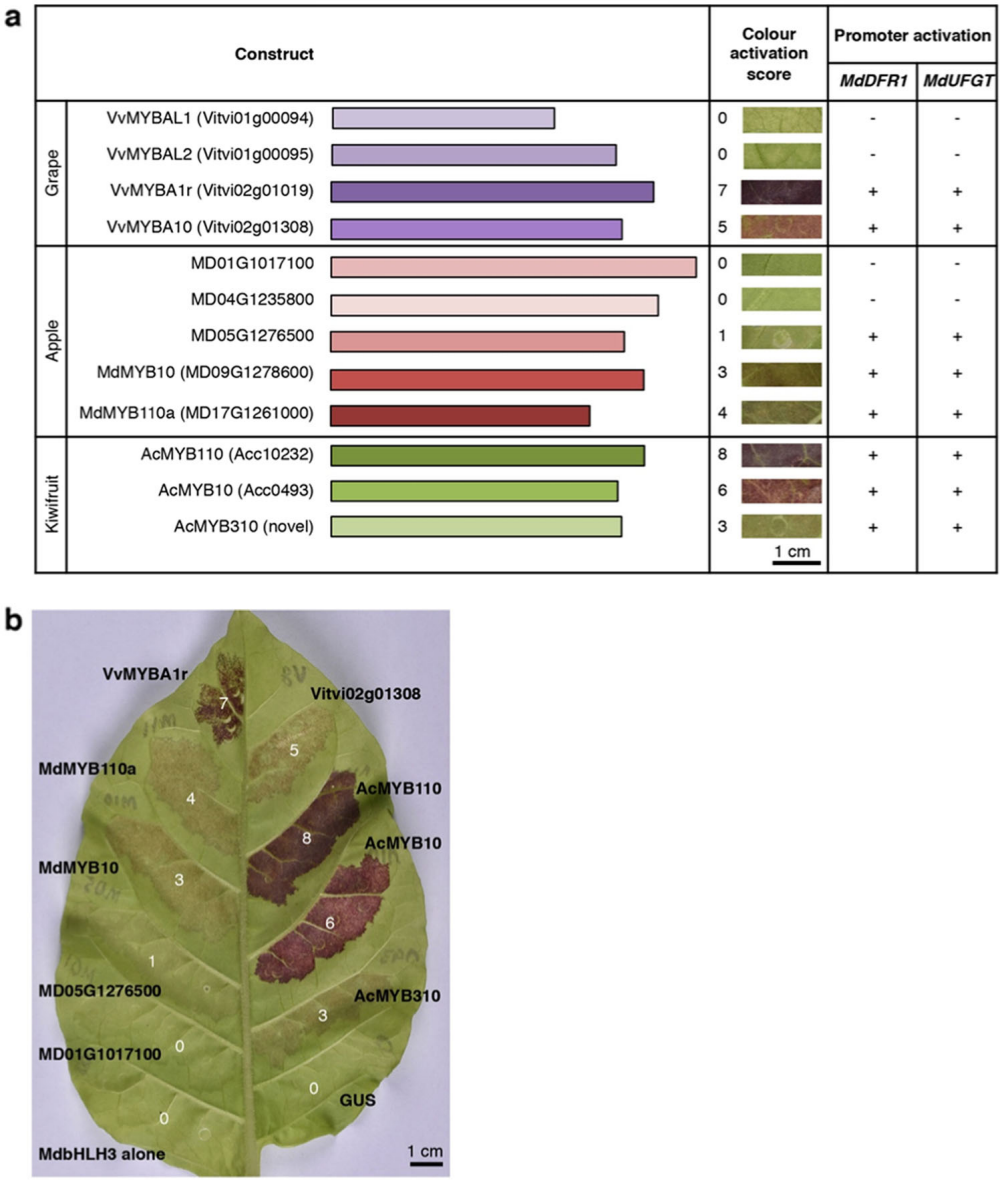
Transient functional assay of select subgroup 6 R2R3 MYB transcription factor genes from apple, grape and kiwifruit. Activation of the endogenous tobacco anthocyanin pathway to produce leaf colouration was tested in *Nicotiana tabacum* and assigned a score from 0 to 8 (**a**, **b**), while qualitative activation of apple anthocyanin pathway promoters of apple *dihydroflavonol-4-reductase 1* (*MdDFR1*) and apple *UDP-glucose:flavonoid 3-O-glycosyltransferase 1* (*MdUFGT1*) was tested by dual-luciferase assay in *Nicotiana benthamiana* (**a**). Both assays involved *Agrobacterium*-mediated infiltration of leaves with apple *MdbHLH3* as a co-factor in addition to endogenous tobacco basic helix–loop–helix (bHLH) transcription factors, followed by measurement after 4 days for *N. benthamiana* and 7 to 12 days for *N. tabacum*. Infiltrated patches of the same construct varied from leaf to leaf and a single representative patch imaged on the abaxial surface is shown with 1-cm scale bars

A third clade of grape MYBs on chromosome 1, consisting of *Vitvi01g00094* (*VvMYBAL1)* and *Vitvi01g00095 (VvMYBAL2*), is the earliest diverging of all the subgroup 6 members analysed. Although VvMYBAL1 and VvMYBAL2 have been previously assigned to subgroup 6, their function remains unclear^35^. Another early diverging MYB is the newly identified apple MYB MD04G1235800. Like VvMYBAL1 and VvMYBAL2, MD04G1235800 clades separately form MYBs with demonstrated anthocyanin-promoting function such as MdMYB10 (MD09G1278600)^41^ and MdMYB110a (MD17G1261000)^42^. We included these genes in transient expression assays in tobacco leaves that compare function of novel and previously described subgroup 6 MYBs that represent the diversity across grape, apple and kiwifruit (Figs. 3a, b, S4, and S5a-h). MYB function was assessed by two measures: quantitatively as stimulation of anthocyanin production by activation of endogenous pathway genes of *Nicotiana tabacum* (Figs. 3a, Fig. 3, S4), and qualitatively by dual-luciferase assay in *Nicotiana benthamiana* to test activation of promoters of apple *dihydroflavonol-4-reductase 1* (*MdDFR1*) and apple *UDP-glucose:flavonoid 3-O-glycosyltransferase 1* (*MdUFGT1*), key genes in the late anthocyanin biosynthesis pathway (Figs. 3a,S5a-h). Since some subgroup 6 MYBs from species distantly related to tobacco, such as MdMYB10 ^41^, appear to be unable to partner with endogenous tobacco bHLHs in order to activate gene expression, we included MdbHLH3 in all infiltrations so tested MYBs are likely to find suitable co-factors for activity.

None of the early diverging subgroup 6 MYBs significantly activated either the *N. tabacum* biosynthetic pathway or the *MdDFR1* and *MdUFGT1* promoters. Apple MdMYB10, MdMYB110a and kiwifruit AcMYB10 and AcMYB110 had high activity in both assays, as previously demonstrated^41–43^. VvMYBA1r also returned positive results, as expected^39^. *VvMYBA10* is expressed along with *VvMYBA1r* and/or other MYBs from the chromosome 2 locus during anthocyanin activation^35^; however, it remains unclear whether it promotes anthocyanin production. Our results show that VvMYBA10 does promote anthocyanin, but not as strongly as VvMYBA1r. Similarly, novel kiwifruit MYB AcMYB310 promotes anthocyanin less strongly than AcMYB10 and AcMYB110. Phylogeny suggests that AcMYB310 is the earliest diverging kiwifruit subgroup 6 MYB (Figs. S2, S3). Additionally, *AcMYB110* and *AcMYB210* are tandem duplicates on linkage group 9, while *AcMYB10* is their homeologue on linkage group 1 from a recent whole-genome duplication specific to the kiwifruit lineage^44^. *AcMYB310* on linkage group 27 appears to have a different origin. The two anthocyanin-activating grape loci on chromosomes 2 and 14 also do not appear to be homeologues from the ancient hexaploidization event in a common ancestor of dicots^45^.

*MdMYB10* on apple chromosome 9 is a homeologue of chromosome 17 *MdMYB110a* from a recent whole-genome duplication^34^. Although novel subgroup 6 apple genes *MD01G1017100* and *MD05G1276500* form a well-supported clade with *MdMYB10*, *MdMYB110a* along with their tandem duplicates (bootstrap of 97), *MD01G1017100* and *MD05G1276500* are not homeologues of each other from the recent apple whole genome duplication. MD01G1017100 did not activate either assay for anthocyanin-promoting ability, while MD05G1276500 activated *MdDFR1* and *MdUFGT1* promoters in *N. benthamiana* but only weakly activated anthocyanin production in *N. tabacum*.

### Conserved C-terminal motifs of anthocyanin-activating subgroup 6 members display molecular recognition feature (MoRF)-like characteristics

**Fig. 4:**
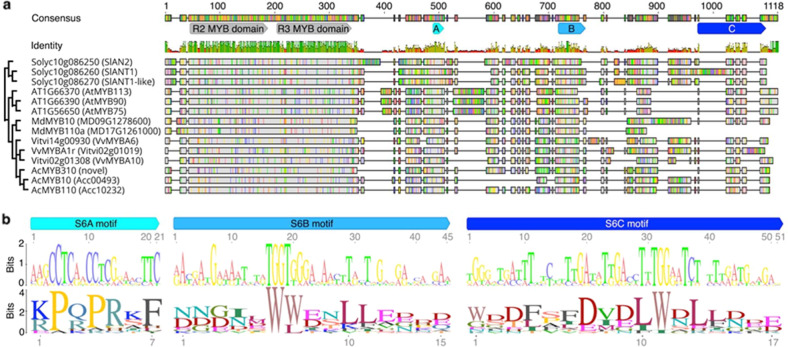
Codon alignment reveals conserved C-terminal sequence motifs of eudicot subgroup 6 R2R3 MYBs that represent the sequence diversity of MYBs with demonstrated anthocyanin activation ability. **a** Codon alignment bases that are identical to the consensus nucleotide are in grey, while those that differ are either in red (adenine), green (thymine), yellow (guanine) or blue (cytosine). Identity is plotted for individual nucleotide positions with green bars indicating 100% identity, yellow bars indicating identity ≥30% but <100 and red bars indicating identity <30%. The dendrogram on the left depicts sequence similarity deduced by the neighbour-joining method. Tandem R2R3 domains are annotated in grey and conserved motifs are in blue, with the previously described subgroup 6 motif^16^ annotated as ‘A’ and two novel motifs annotated as ‘B’ and ‘C’. Previous publications provide evidence for the anthocyanin activation ability of tomato genes *SlAN2*, *SlANT1* and *SlANT1-like*^21,80^, *Arabidopsis* genes *AtMYB113*, *AtMYB90* and *AtMYB75*^61^, apple genes *MdMYB10* ^41^ and *MdMYB110a*^42^, grape genes *VvMYBA6*^40^ and *VvMYBA1r*^39^, and kiwifruit genes *AcMYB10* ^81^ and *AcMYB110* ^82^. **b** Consensus sequence logos of nucleotide (upper panel) and protein (lower panel) alignments of subgroup 6 motifs A, B and C using gene sequences shown in **a**. Consensus alignment gaps were ignored when constructing sequence logos for motif S6C

**Fig. 5:**
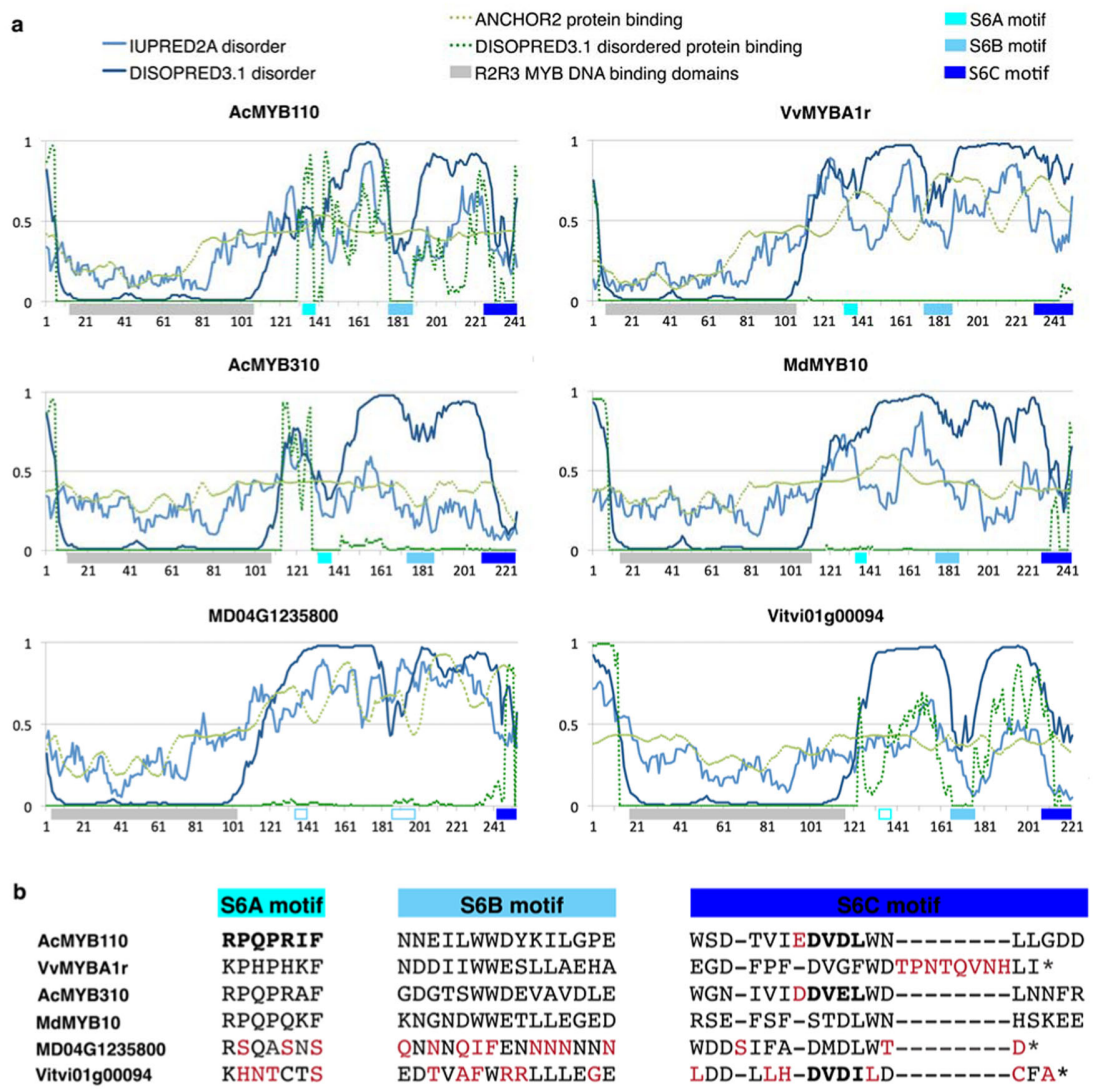
Protein regions corresponding to conserved C-terminal motifs of anthocyanin-activating subgroup 6 R2R3 MYBs are predicted to be less disordered than flanking regions, with non-activators (MD04G1235800, Vitvi01g00094) diverging at S6A and S6B motifs compared with strong anthocyanin activators (AcMYB110, VvMYBA1r) and moderate activators (AcMYB310, MdMYB10). **a** Disorder and protein binding scores along select subgroup 6 R2R3 MYB protein sequences, as predicted by IUPred2A^46^ and DISOPRED3^47^ programs for protein structure analysis. Amino acid position is plotted along the *x*-axis with conserved MYB DNA binding domains and C-terminal motifs denoted by filled grey and blue boxes, respectively. Clear boxes outlined in blue highlight regions that align with the sequence motifs of other MYBs but are considerably diverged from the consensus. Predicted disordered regions are defined by a disorder score of 0.5 to 1, while protein binding regions are defined by a binding score of 0.5 to 1. Note that, unlike ANCHOR2, DISOPRED3.1 specialises in predicting protein binding probability for disordered regions and does not make predictions for ordered regions. **b** A representative sample of sequence variation at C-terminal motifs within subgroup 6. Amino acids are shown in red when they differ from the activator-based consensus motif and bold when part of a perfect match to a Eukaryotic Linear Motif database entry (DOC_MAPK_MEF2A_6 for S6A and LIG_WD40_WDR5_VDV_2 for S6C)

The C-terminus of subgroup 6 members is more divergent than the R2 and R3 DNA binding domains (Fig. S6), and the extent to which this variation correlates with variation in activation strength is unclear. To identify functional C-terminal regions, we first searched sequence conservation among MYBs that moderately or strongly activated anthocyanin (Fig. 4). We defined moderate to strong activation as a colour development score >1 in our study or similar colour development in the literature. Three regions were identified of 15 to 17 codons that were more conserved than average, and these were named S6A, S6B and S6C. As the S6A region encompassed the previously described 7-codon motif that characterises subgroup 6, we equated the S6A SliM with the previously termed “subgroup 6 motif”^16^. IUPred2A^46^ and DISOPRED3^47^ were then used to predict protein disorder and protein-binding potential across anthocyanin activating and non-activating MYBs (Figs. 5, S5i). Results indicate that the highly conserved seven amino acids of the S6A motif as well as the core five to eight amino acids of S6B and S6C motifs possessed a greater tendency toward structural order than flanking intrinsically disordered regions (Figs. 5, S5i), a characteristic of MoRFs^10^. Additionally, both S6B and S6C motifs were enriched in hydrophobic and acidic residues and localised near the C-terminus in at least one MYB.

It is interesting that the S6A motif, which has a confirmed role in post transcriptional repression^25^, also demonstrated MoRF-like features which are functional in the protein. The Eukaryotic Linear Motif (ELM) database^48^ lists the similar SliM [RK]-x(2,4)-[LIVMP]-x-[LIV]-x-[LIVMF] (ELM identifier: DOC_MAPK_MEF2A_6; described here in PROSITE notation) to be a kinase docking motif that mediates interaction with the ERK1/2 and p38 subfamilies of MAP kinases. *Arabidopsis* MAP KINASE4, which belongs to the ERK subfamily, stabilises subgroup 6 MYB AtMYB75 and is essential for light-induced anthocyanin accumulation^49^. In line with the hypothesis that S6A variation fine-tunes an interaction with MAP KINASE4, AcMYB110 is the only MYB to fully match the ELM database DOC_MAPK_MEF2A_6 motif with its sequence R-P-Q-P-R-I-F-M (Fig. S6), and was also the strongest activator in our assay.

Like the S6A motif, S6B did not fully match any known ELM database entries, but its core SLiM is similar to the SLiM x-x-x-x-W-F-x-x-L (ELM identifier: LIG_PALB2_WD40_1) required by the BRCA2 DNA repair protein for binding to WD40 protein PALB2. The core SLiM of S6C was the only one to fully match an ELM database entry: [EDSTY]-x(0,4)-[VIPLA]-[TSDEKR]-[ILVA] (ELM identifier: LIG_WD40_WDR5_VDV_2). This is a fungi-specific motif that binds to WD40 protein WDR5, which mediates histone modification complex assembly. S6C also shares similarities with [ED]-x(0,3)-[VIL]-D-[VI] (ELM identifier: LIG_WD40_WDR5_VDV_1; the metazoan version of LIG_WD40_WDR5_VDV_2), and F-[EDQS]-[MILV]-[ED]-[MILV]-((x(0,1)-[ED]) | (*)) (ELM identifier: DOC_WD40_RPTOR_TOS_1; occurs either at the N- or C-terminal end and binds to the WD40 protein Raptor, which is a part of the TOR protein kinase complex). In general, MYBs with no or poor anthocyanin-promoting ability possessed distinct sequence variation at S6A, S6B and S6C regions compared with stronger activators (Figs. 5, S6), but this was most pronounced at S6A.

### Engineering of motifs from weak anthocyanin activators into strong anthocyanin activators and vice versa reveals functional effects of sequence variation

**Fig. 6:**
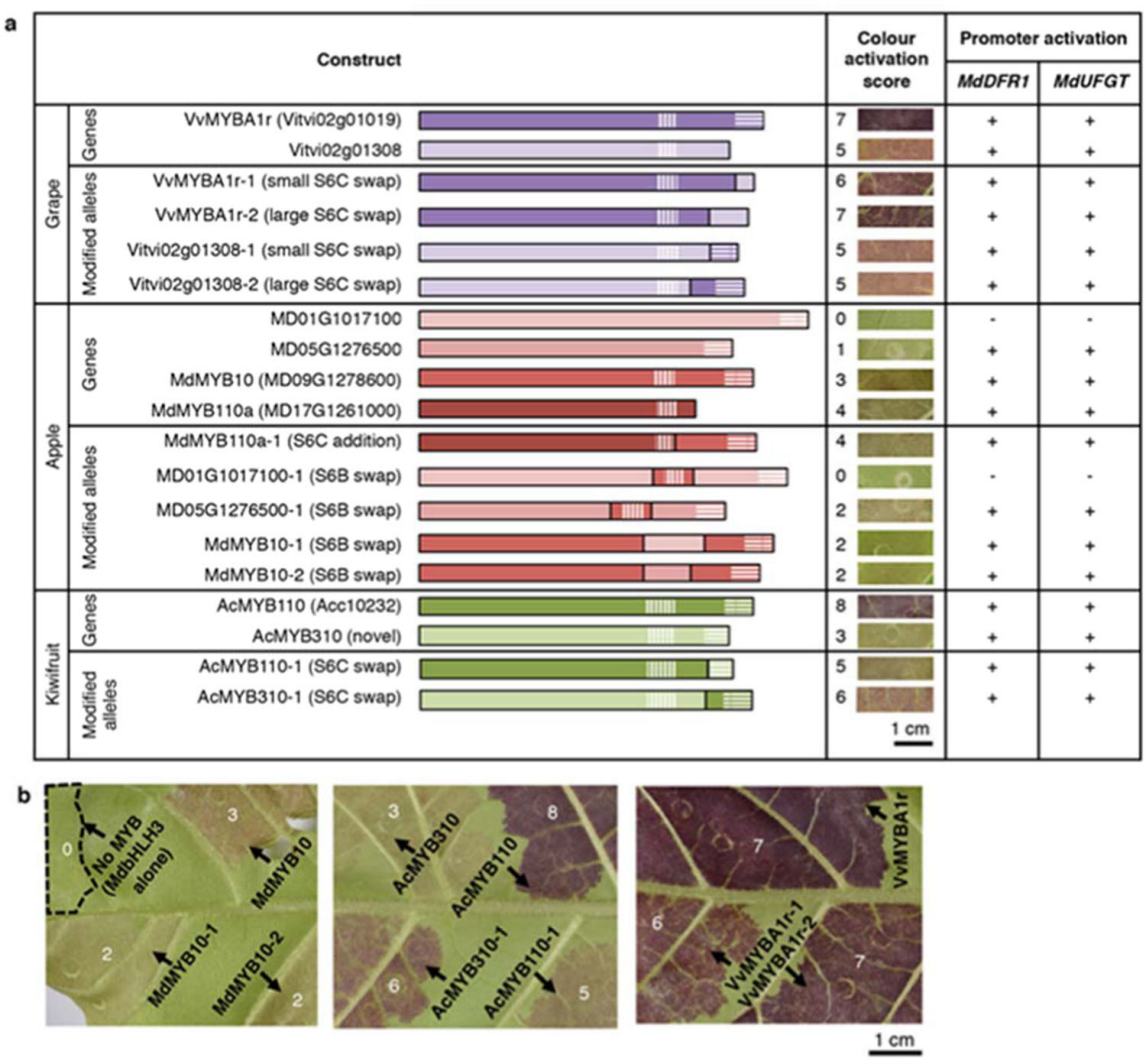
Transient functional assay of natural and modified alleles of subgroup 6 R2R3 MYB transcription factor genes from apple, grape and kiwifruit in tobacco leaves. Activation of the endogenous tobacco anthocyanin pathway to produce leaf colouration was tested in *Nicotiana tabacum* and assigned a score from 0 to 8 (**a**, **b**), while qualitative activation of apple anthocyanin pathway promoters of apple *dihydroflavonol-4-reductase 1* (*MdDFR1*) and apple *UDP-glucose:flavonoid 3-O-glycosyltransferase 1* (*MdUFGT1*) was tested by dual-luciferase assay in *Nicotiana benthamiana* (**a**). Both assays consisted of *Agrobacterium*-mediated infiltration of leaves with apple *MdbHLH3* as a co-factor in addition to endogenous tobacco basic helix–loop–helix (bHLH) transcription factors, followed by measurement after 4 days for *N. benthamiana* and 7 to 12 days for *N. tabacum*. Transfer of the S6B motif sequences (vertical hatching) or S6C motif sequences (diagonal hatching) between closely related genes that differ in anthocyanin activation strength demonstrates that both regions contribute to overall activation strength of individual transcription factors. Infiltrated patches of the same construct varied from leaf to leaf and a single representative patch imaged on the abaxial surface is shown with 1-cm scale bars

As the functional importance of S6A is already underscored by its role as a small RNA target site, we focused on testing whether S6B and S6C motifs contributed to MYB function. Unlike for subgroup 12, subgroup 6 C-terminal motifs are not expected to play a role in bHLH interaction, since the C-terminus of subgroup 6 AtMYB75 cannot recruit bHLH AtGL3, while that of subgroup 12 AtMYB29 recruits bHLH AtMYC4^8^. Eleven modified gene sequences were created by swapping motif sequences between closely related subgroup 6 MYBs that varied in colour activation and sequence (Fig. 6a, Supplementary Materials and Methods). For most motif swaps, flanking sequences were included on either side of the motif, as they also play a role in defining specificity and affinity of MoRF interactions^10,12^.

The modified MD05G1276500-1 allele, where the natural S6B sequence is replaced with that of stronger anthocyanin activator MdMYB10, showed greater anthocyanin production than the un-modified allele in *N. tabacum* leaves, with a score of 2 versus 1 (Figs. 6a, S4). The insertion of MdMYB10 S6B sequence into a modified MD01G1017100 allele did not increase colour development from the initial score of 0, but this was probably due to the effect of mutations in the R2R3 DNA binding domain of MD01G1017100 that cannot be ameliorated by an improved S6B region. MD01G1017100 contains several non-synonymous mutations in the MYB DNA binding domains that are not shared by any anthocyanin-activating MYBs: R17K, R24Q, Q25H, C26F, K29Q, H36R, T69G, I77V, L83V, L89M and N107S (Fig. S6). Replacement of the MdMYB10 S6B motif with S6B motifs from either MD01G1017100 or MD05G1276500 decreased the colour activation score from 3 to 2 (Figs. 6a, b, S4), supporting the hypothesis that this region fine-tunes the activity of the MYB.

For the S6C motif, replacement of the strong activator AcMYB110 motif sequence into the weaker AcMYB310 improved anthocyanin activation in AcMYB310-1, while engineering of the weak AcMYB310 S6C motif into AcMYB110 decreased anthocyanin activation in AcMYB110-1. Although MdMYB110a is a naturally truncated allele, where a frameshift mutation soon after S6B produces a premature termination codon with loss of S6C, it is a stronger anthocyanin activator than MdMYB10, which contains both S6B and S6C motifs. Addition of the MdMYB10 S6C motif to MdMYB110a did not further increase colour activation in MdMYB110a-1. This suggests that the MdMYB10 S6C motif is ineffective in the assay or functionally redundant to at least one other motif of MdMYB110a, probably the S6B motif given sequence similarly. Vitvi02g01308 is also mutated near the C-terminus with apparent loss of S6C. However, although the sequence replacing S6C in Vitvi02g01308 does not match the WDR5-binding motif and differs from the TOS motif by substituting acidic resides [ED] with G, it is similar to S6C in being enriched for acidic and hydrophobic residues and is predicted to be even more ordered than the VvMYBA1r S6C motif (Fig. S6, S5i). For VvMYBA1r and Vitvi02g01308, we tested the effect of swapping either a smaller fragment that included the core S6C SLiM but excluded flanking sequence or a larger fragment that included 13 to 21 codons of flanking sequence. Neither size of the VvMYBA1r sequence swapped into Vitvi02g01308 improved anthocyanin production, just as when the larger Vitvi02g01308 sequence was swapped into VvMYBA1r. However, when the smaller Vitvi02g01308 S6C sequence was swapped into VvMYBA1r, colour activation score decreased from 7 for VvMYBA1r to 6 for VvMYBA1r-2. This highlights how flanking disordered sequences are important for interaction at SLiMs^10,12,13^.

## Discussion

### Codon models and improved genomic resources enhance phylogenetic analyses

This work builds upon previous studies which explored MYB diversity by harnessing recent improvements to reference genomes and increased availability of gene expression data. Codon models of evolution outperform nucleotide and amino acid models in phylogenetic analysis^36,37^, and our codon model-based phylogeny better resolves relationships between MYB subgroups. The topological improvements made by sampling genes from an additional species, tomato, and excluding non-functional proteins, are evident by comparison of the two different trees in the analysis. Some well-supported conclusions of our phylogenetic analysis have been indicated with weaker support in other phylogenies, including those built with multiple plant species^3,50^ and species-focused phylogenies for grape^35^ and kiwifruit^30^. These comprise the early divergence of R2R3 subgroups 29 (CDC5-like), 28 (AtMYB88, AtMYB124) and 25 relative to subgroups that expanded during the transition to life on land, and the early divergence of subgroups 18, 22, 23, 26 and 27 within the larger clade of land plant-specific subgroups. The indication that most land plant-specific MYB regulators of secondary metabolism evolved from an ancient algal regulator of growth and development may be unsurprising given that development-related subgroup 15 is the closest sister to anthocyanin-regulating subgroup 6.

The placement of subgroups 4, 19 and 20 as the closest relatives of the flavonol- and anthocyanin-regulating clade is mirrored in multi-species phylogenies of *Arabidopsis*, peach, strawberry and apple^31^, as well as single-species phylogenies of *Arabidopsis*^7^ and grape^35^. However, multi-species phylogenies that span land plants^3,50^ place development- and stress-related subgroups 19 and 20 closer to cell wall metabolism-related subgroups 13 and 16, although with lower support than our analysis. At this stage it is unclear if this latter result is due to increased taxon sampling, reducing the effect of homoplasy^38^, or whether codon models more effectively resolve deep divergence. In support of our placement, the subgroup 20 MYB AtMYB112 shares similarities with subgroup 6 members^51^ in promoting anthocyanin formation under abiotic stress and specifically activating subgroup 4 repressor MYBs AtMYB6, AtMYB7 and AtMYB32^52^. If true, this placement would be another indication of a short evolutionary distance between secondary metabolite regulation and developmental regulation. Most previous publications support our clading of repressor MYB subgroup 4 with flavonol and anthocyanin regulators^7,31,35,50^, suggesting that repression evolved alongside activation from a common ancestral subgroup.

### C-terminal S6A, S6B and S6C motifs of subgroup 6 MYBs are putative MoRFs, suggesting roles in protein–protein interactions

Each relatively conserved region within the C-terminus (i.e. S6A, S6B and S6C) was also predicted to have greater protein structural order than the rest of the disordered C-terminus. This suggests they are MoRFs, which are often disordered when isolated but form secondary structures upon interaction^11^. Subgroup 6 MYBs, like many other environmentally responsive TFs, can participate in large interactomes, and this is facilitated by having intrinsically disordered protein regions that allow flexible interaction^12,13^. Disorder predictions varied between DISOPRED3 and IUPred2A at times, possibly owing to differences in algorithm and datasets used to train the predictors via machine learning.

The implication that motif S6A plays a role in regulation by TAS4 as well as in protein–protein interaction, is not only supported by our analysis of predicted structure but also by a study that found protein sequence in tobacco and *Arabidopsis* was conserved better than DNA sequence despite eventual mismatches to TAS4-siRNA81(-)^25^. Evolution may favour the overlap of small RNA target sites with regions of conserved protein function, as seen in NB-LRR genes^53^. Additionally, many subgroup 6 MYBs are the targets of the microRNAs miRNA-858 and miRNA-828 at sites in the R2R3 MYB DNA binding domain important for protein function^24,54^. The similarity between S6A and ELM database^48^ entry DOC_MAPK_MEF2A_6, along with the previous finding that *Arabidopsis* MAP KINASE4 stabilises subgroup 6 MYB AtMYB75 and is essential for light-induced anthocyanin accumulation^49^, implies a role for S6A in interaction with MAP KINASE4. Our transient assays in tobacco leaves were strongly influenced by the presence of light, possibly accounting for S6A variation being a greater predictor of activation strength than that at S6B and S6C. The discrepancy between the S6A consensus and DOC_MAPK_MEF2A_6 may be due to a lack of plant instances for this motif in the ELM database. MoRFs conserved across biological kingdoms have more greatly diverged instances among phyla and subphyla than do ordered protein domains, so divergence between plant and mammalian instances is expected^11,14^.

Core SLiMs of S6B and S6C motifs differed from those of S6A, being enriched in hydrophobic and acidic residues and matching a generic SLiM Φ-x-x-Φ-Φ (where Φ indicates a hydrophobic residue) seen in the activation domain of many TFs^13^. S6B is conserved in *Antirrhinum* subgroup 6 MYBs^55^, while an S6C-like motif is required for activity of maize subgroup 5 anthocyanin activator ZmC1^56^. Subgroup 6 MYBs show no or severely decreased activity after loss of S6B and S6C in *Petunia*^57^, or even S6C alone in *Petunia* and *Trifolium*^58^. Sequence variation at and near the ZmC1 S6C-like motif alters activation strength^59^, as does that at a similar essential SliM of plant AP2/ERF TFs^60^. We show the functional importance of S6B in motif swaps between apple MYBs. The S6B SLiM is similar to ELM database entry LIG_PALB2_WD40_1, the motif of DNA-binding protein BRCA2 that binds WD40 protein PALB2. Although functional differences between BRCA2 and subgroup 6 MYBs make it unlikely that S6B recruits a plant PALB2 homolog, it is likely that another WD40 protein plays a role. Many anthocyanin-activating MYBs require the presence of a WD40 scaffold protein along with a bHLH cofactor to upregulate target anthocyanin biosynthetic enzymes like DFR, UFGT, and leucoanthocyanidin dioxygenase (LDOX; also known as anthocyanidin synthase or ANS)^22,61^, with the WD40 partner undiscovered in many cases^5^. The S6C motif also matched ELM database motifs for interaction with WD40 proteins, although it is possible that putative WD40 interactors include WDR5 and key proteins of the TOR protein kinase complex, since some of their functions appear to be conserved between metazoans, fungi and plants^62–65^ and appear to affect anthocyanin accumulation^66^. Whilst several SLiM-binding domain families are experimentally verified to bind non-canonical motifs, WD40 family members are especially known to target physicochemically diverse motifs, possibly explaining why the S6C replacement sequence in Vitvi02g01308 sustains function^12^. As S6C sequence variation has a strong effect in the motif swap between AcMYB110 and AcMYB310, it is likely that S6C loss in MdMYB110a and S6C swaps in grape were ineffectual because S6C function is redundant to that of S6B, or our assay was less suitable for apple and grape MYBs than for kiwifruit MYBs. SLiM redundancy has been previously described for mammalian proteins^13^ and species-specific bias of the tobacco leaf assay is highlighted by increased interaction of kiwifruit MYBs with endogenous tobacco bHLHs^43^ compared with MYBs from apple, which is a more distantly related species^41,67^.

Notably, S6B and S6C motifs are similar in relative location and sequence to previously described motifs for closely related subgroups 15 (W-V-x-x-D-x-F-E-L-S-x-L) and 5 (D-E-x-W-R-L-x-x-T), respectively^16^. The subgroup 15 motif of Arabidopsis WEREWOLF is sufficient for transcriptional activation in yeast one-hybrid assay^68^, but its loss in an allele of Arabidopsis GLABROUS 1 (AtGL1) appears to be partially rescued by fellow subgroup 15 member AtMYB23 as well as AtGL1 interaction with the bHLH GLABROUS 3 (AtGL3) that recruits WD40 protein TRANSPARENT TESTA GLABRA 1 (AtTTG1)^69^. Like subgroup 6, subgroups 15 and 5 function in complex with bHLH and WD40 cofactors^5^, with bHLH interaction occurring through the MYB R3 sequence^20^. Subgroup 15 and 5 motifs probably recruit regulators similar to those recruited by S6B and S6C, thereby affecting transcriptional activation strength. C-terminal motifs enriched in hydrophobic and acidic residues also occur at similar positions in widely diverged subgroups 1 to 6, 15, 16 and 21^16^. Motifs for subgroups 9, 11 and 19 are also enriched in hydrophobic and acidic residues but positioned more similarly to S6A^16^. Other subgroups may possess homologous or analogous regions that are difficult to detect owing to the rapid evolution of SLiMs, as seen by comparing the S6C motif of VvMYBA1r with the equivalent region in Vitvi02g01308.

### S6A, S6B and S6C motifs provide opportunities for dissecting and harnessing MYB function

Although sequence variation within MoRFs is well tolerated, evidence suggests that the sequence within and also flanking interaction sites fine-tunes interaction strength^12,13^. Future work aimed at characterising interactors of S6A, S6B and S6C motifs will elucidate regulatory mechanisms that are conserved across eudicot MYB subgroup 6 members and possibly even across eudicot MYB subgroups. It is possible that S6A, S6B and S6C contain further overlapping SLiMs undetected in our analysis. Experimental validation of SLiMs will help build valuable resources for the detection of plant SLiMs. This provides opportunities for both engineering MYB TFs with optimal function as well as sequence-based germplasm screening to identify ideal lines for breeding elite crops.

## Materials and methods

### Genomic analysis

Gene annotation employed the *Actinidia chinensis* Red5 kiwifruit genome^44^, doubled haploid *Malus* x *domestica* ‘Golden Delicious’ apple genome^34^ and updated 12xV3 version of the *Vitis vinifera* “Helfensteiner x Pinot noir” grape genome^45,70^. The method summarised in Fig. 1 is detailed in the Supplementary Materials and Methods. *Arabidopsis* and tomato transcripts were obtained from the TAIR10 ^27^ and ITAG4.0^26^ annotations, respectively. Gene products were scanned for the MYB Hidden Markov Model (PF00249) using hmmer version 3.1b2 (http://hmmer.org). R2R3 and 3R MYBs were identified by the presence of tandem MYB domains. Two iterative BLAST^71^ searches detected R2R3 and 3R MYBs that failed screening with PF00249, as well as single domain R3 MYBs. The resultant list was manually curated to exclude MYB-related genes and correct instances where MYB gene annotations were fused with that of another gene.

### Phylogenetic analysis

To infer the R2R3 MYB phylogeny, we only used tandem R2R3 domain sequences free of frameshift mutations and premature termination codons. For our codon-model analysis, codons of 846 functional R2R3 and 3R MYBs from apple, *Arabidopsis*, grape, kiwifruit and tomato were aligned with MACSE v2^72^ and manually refined in Geneious 10.0.9 (https://www.geneious.com) to generate a 336 nucleotide-long alignment after masking non-informative positions. A maximum likelihood phylogeny with 10,000 bootstrap replicates under the MGK + F3X4 + R9 codon model of evolution was generated with iqtree version 1.6.11 using the command “iqtree -s alignment.phy -st CODON -nt 1 -seed 5385 -bb 10000 -bnni”^73–76^. A second maximum likelihood phylogeny was generated with RAxML 8.2.11^77^ using a 221 amino acid-long alignment of 791 R3, R2R3 and 3R MYBs of apple, *Arabidopsis*, grape and kiwifruit and the JTTDCMUT substitution model. The command used was “raxmlHPC -f a -x 621986 -p 621986 -N autoMRE -m PROTGAMMAAUTO”. The amino acid alignment was generated by manual refining an alignment with default settings of the MAFFT^78^ plug-in in Geneious 10.0.9 (https://www.geneious.com). Informative positions are provided in the alignments in Supporting information Datasets S1 and S2. MYB subgroup membership was inferred phylogenetically, with previously published information on MYB diversity of algae and early diverging land plants^3,50^ serving to identify ancient subgroups and orient the unrooted trees.

### Characterisation of C-terminal sequence motifs

Full-length nucleotide sequences of novel and previously described MYBs that represent the sequence diversity of subgroup 6 were aligned using MACSEv2 ^72^ with manual refinement in Geneious 10.0.9. Conserved regions were detected based on identity across the alignment and motif sequence logos were generated with Geneious. Protein disorder was predicted with IUPred2A^46^ and DISOPRED3^47^ under default settings.

### Generation of MYB constructs

Constructs for AcMYB10, AcMYB110, MdMYB10 and MdMYB110a were as produced previously^43,67^. Other subgroup 6 MYBs were synthesised into a pUC57-Kan plasmid with flanking attL sites (Genewiz^®^ LLC, genewiz.com) based either on the sequences of de novo assembled transcripts or that of the reference genome if not significantly different from de novo assemblies: constructs referred to primarily by annotation number derived from the reference genome, while those referred to by MYB number originate in a different cultivar. Naturally occurring BsaI sites were mutated to facilitate Golden Gate cloning of motifs of weak activators into strong activators and vice versa. Cloning methods are detailed in the Supplementary Materials and Methods, with the Golden Gate cloning approach following recommendations of the laboratory of Jeffrey Barrick at The University of Texas at Austin (https://barricklab.org/twiki/bin/view/Lab/GoldenGateAssemblyProtocolsMainPage).

### Dual-luciferase assay

The ability of MYB constructs to activate the promoters of apple anthocyanin pathway genes *MdDFR1* and *MdUFGT1* was tested in the presence of MdbHLH3 as a co-factor by a previously described dual-luciferase assay involving transient *Agrobacterium*-mediated transformation of *Nicotiana benthamiana* leaves^79^. Details are in the Supplementary Materials and Methods.

### Transient colour development assay in *Nicotiana tabacum*

The ability of MYB constructs to activate endogenous anthocyanin biosynthesis in *Nicotiana tabacum* was tested by transient *Agrobacterium*-mediated transformation of MYB constructs into growing leaves along with the co-factor MdbHLH3, as previously described^41^. *Agrobacterium tumefaciens* GV3101 transformants for *MdbHLH3* and subgroup 6 MYB genes were grown, prepared and infiltrated into leaves as described for the dual-luciferase assay, with the exclusion of dual-luciferase constructs from the final inoculation mixture. Leaves from *N. tabacum* were used, as *N. bentham*iana does not produce anthocyanins naturally. Colour development was assayed between 7 and 12 days after infiltration (DAI). Constructs were compared with positive and negative controls on the same leaf, and colour activation scores ranging from 0 to 8 were assigned on the basis of at least 10 biological replicates.

## Supplementary information

Supplementary Information

Fig S2

Fig S3

Fig S6

Datasets 1-3

## Data Availability

In addition to inclusion of sequences used for phylogenetic analysis in Datasets S1 and S2, genomic locations of all apple, grape and kiwifruit MYBs, including novel MYBs identified by genomic analysis in this study, are released in Dataset S3. The nextflow pipeline for automated genome annotation enhancement is provided in the Supplementary Information as Code S1.
