# Supplementary material for: Genomic analysis uncovers functional variation in the C-terminus of anthocyanin-activating MYB transcription factors
Source: Hortic Res. 2021 Apr 1;8:77. doi: 10.1038/s41438-021-00514-1 (PMC8012628; doi:10.1038/s41438-021-00514-1)
Supplement: Supplementary file 1 — Supplementary Information [file 41438_2021_514_MOESM1_ESM.docx]

**Genomic analysis uncovers functional variation in the C-terminus of anthocyanin-activating MYB transcription factors**

**Supplementary Information**

Supplementary Figures S1-S6 (S2, 3 and S6 provided as separate files)

Supplementary Materials and Methods

Supplementary Data Sets S1-S3 (provided as separate files)

Supplementary Tables S1-S3

Supplementary Code S1

**Supplementary Figures**


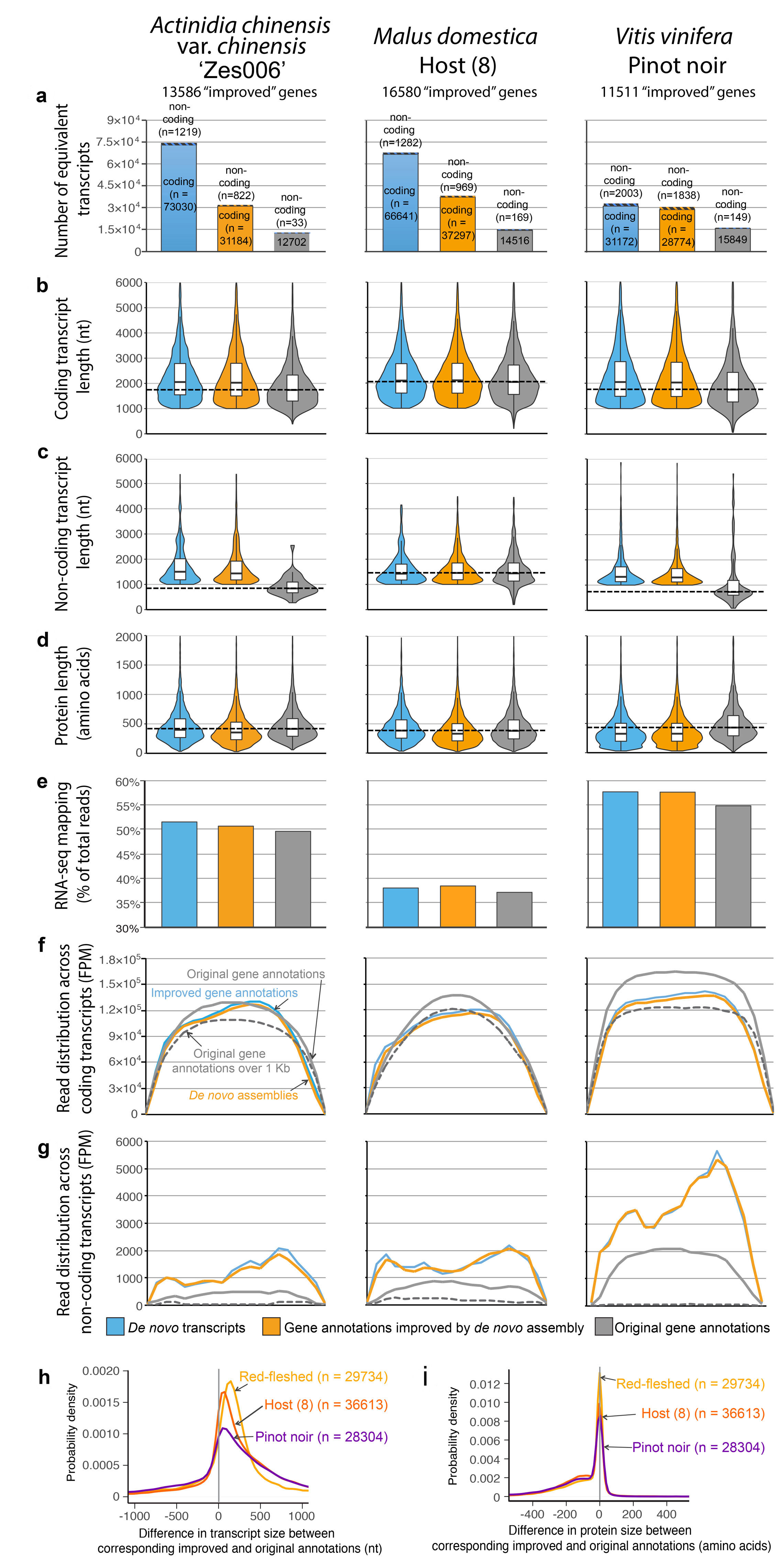


**Figure S1. Genome annotation guided by *de novo* transcript assemblies better characterises non-coding sequences (e.g. untranslated mRNA regions and long non-coding RNAs), and can be as descriptive as *de novo* assemblies themselves.** To specifically assess the potential of *de novo* transcriptomes to improve genome annotations, comparison was made only among improved gene annotations, the *de novo* transcripts (size ≥1Kb) that produced them, and the published gene annotations that they overlapped. **a** Transcripts were divided into non-coding and coding groups, with non-coding transcripts defined as those encoding fewer than 100 amino acids and not sharing >50% overlap with a coding transcript. Note that improved annotations include more differentially spliced transcripts. **b,c,h** On average, *de novo* assembled transcripts and improved annotations derived from them were longer than published annotations. Dotted lines in (**b,c,d**) indicate the median for published annotations. **d,i** Average protein size of published annotations was greater than that for *de novo* transcripts and improved annotations, owing to both under-representation of long non-coding RNAs and a decrease in coding length for a small subset of improved annotations (**i**). The decrease in (**i**) may result from intron retention in alternatively spliced transcripts of improved annotations. **e** For all cultivars, more RNA-sequencing (RNA-seq) reads mapped to *de novo* transcripts and improved annotations than to published annotations. **f,g** RNA-seq read distribution across transcripts revealed the expected slight 3’ bias owing to poly-A selection and cDNA fragmentation in *de novo* and improved subsets, but not in published subsets. This is probably due to inaccurate definition of transcript termini in published subsets. Abbreviations: nt, nucleotides; Kb, kilobases

**Figure S2. Maximum likelihood phylogeny of 846 functional R2R3 and 3R MYBs of *Arabidopsis*, apple, grape, kiwifruit and tomato used in built using iqtree software.** (provided as a separate file)

**Figure S3. Maximum likelihood phylogeny of 791 functional and non-functional R3, R2R3 and 3R MYBs of *Arabidopsis*, apple, grape, and kiwifruit built using RAxML software.** (provided as a separate file)


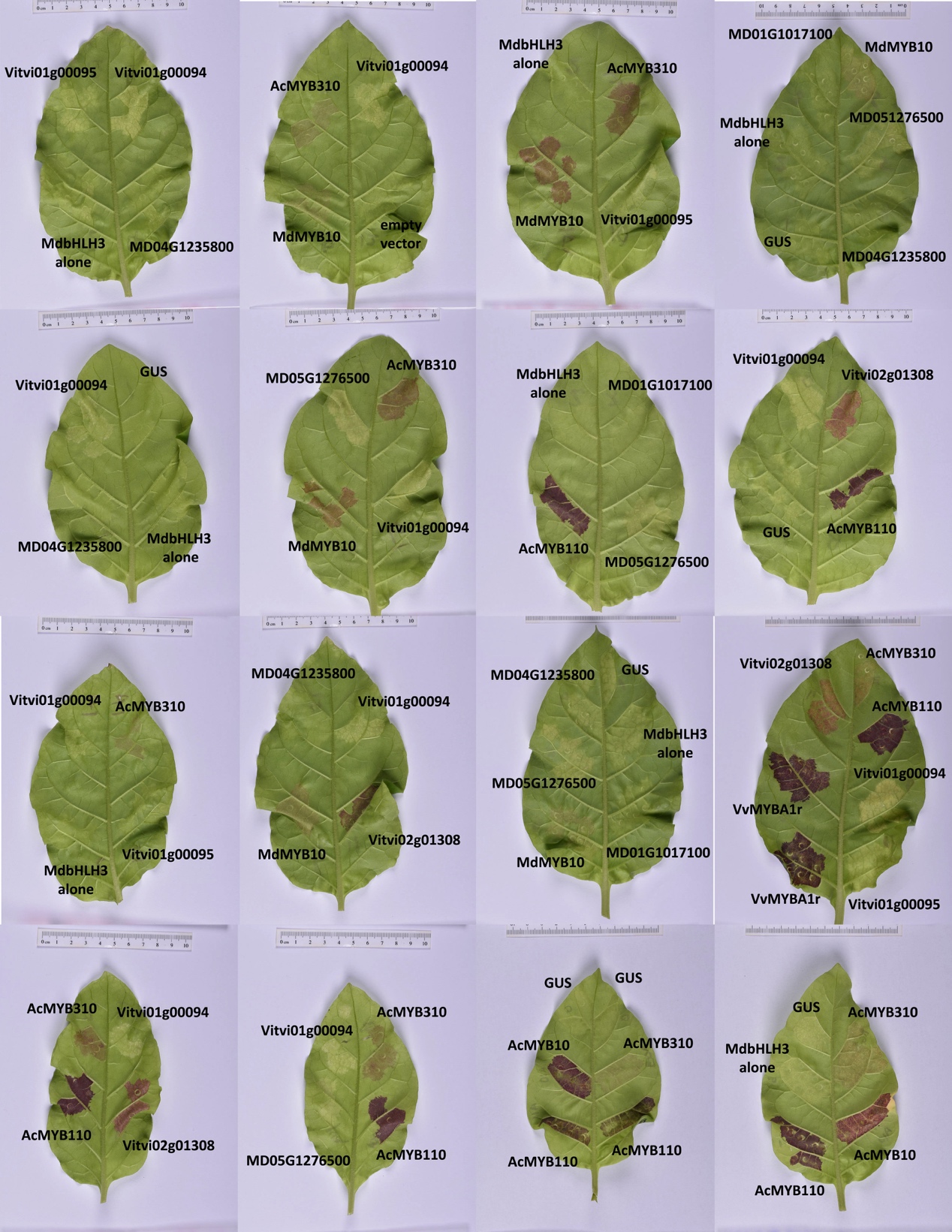


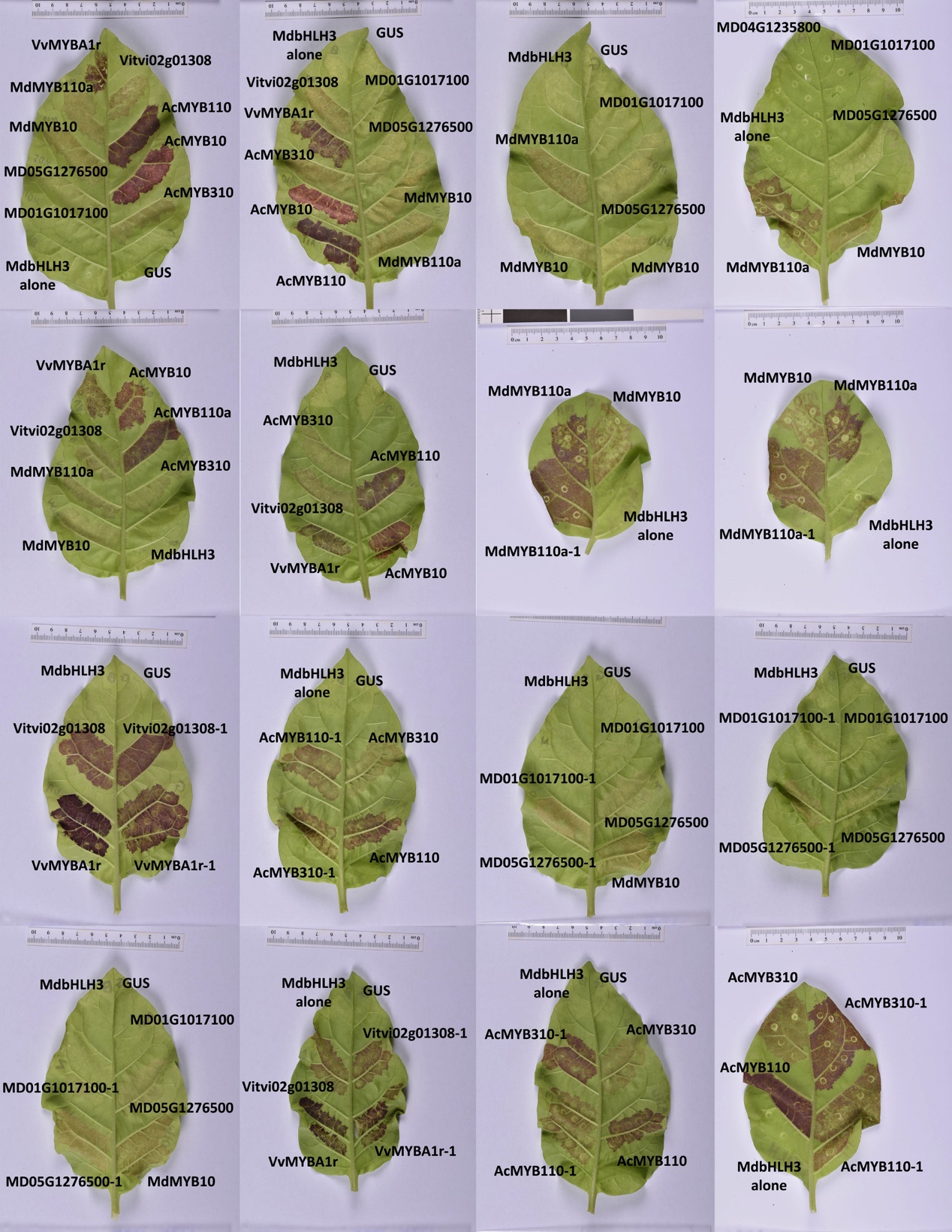


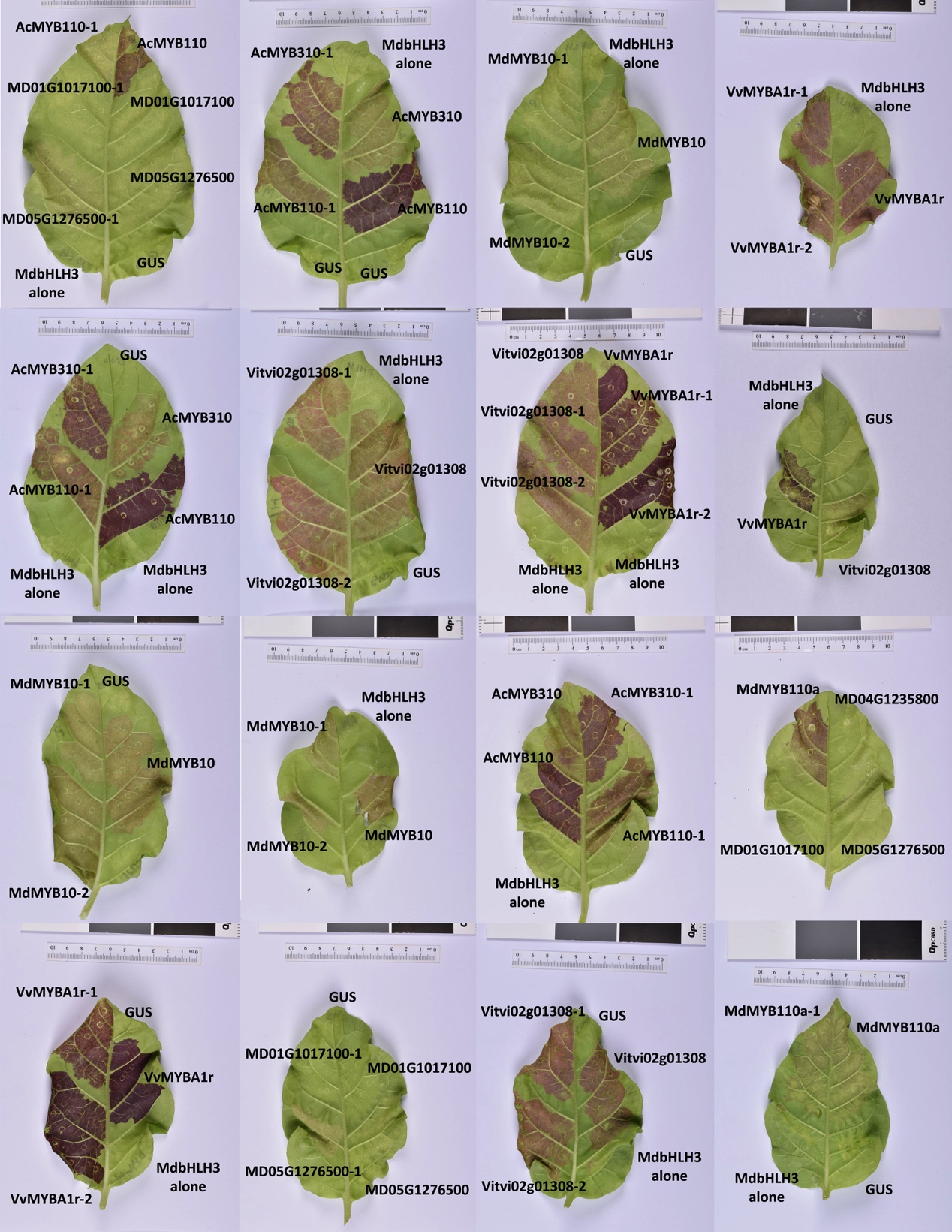


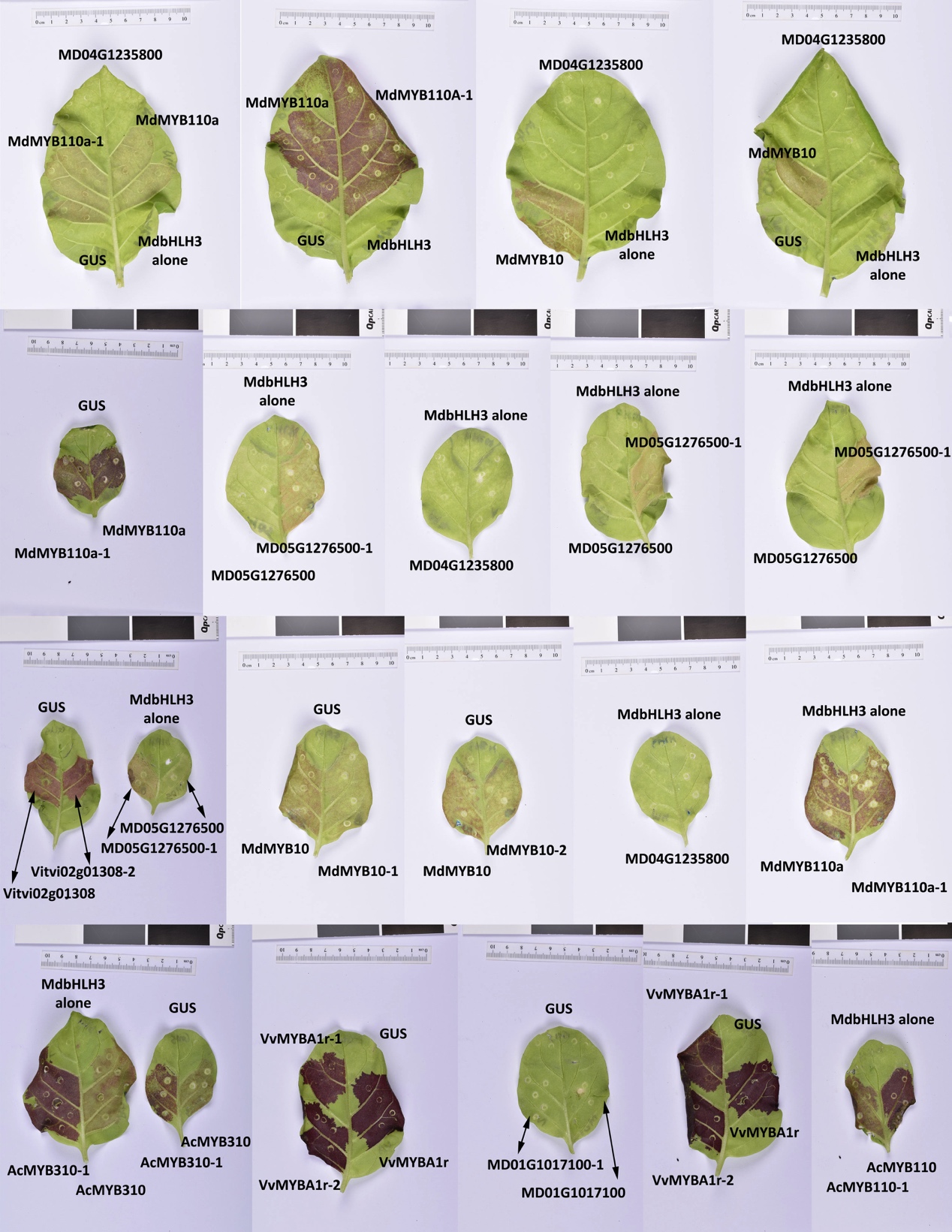


**Figure S4. Activation of endogenous colour-producing pathways in *Nicotiana tabacum* leaves by transient expression of natural and modified alleles of subgroup 6 R2R3 MYB transcription factor genes from apple, grape and kiwifruit.** The assay consisted of *Agrobacterium*-mediated infiltration of leaves with apple *MdbHLH3* as a co-factor in addition to endogenous tobacco basic helix-loop-helix (bHLH) transcription factors, followed by measurement after 7 to 12 days. All images are taken on the abaxial leaf surface and are shown at the same scale, with the ruler showing measurements in centimetres. Note that infiltrated patches of the same construct varied from leaf to leaf, so colour activation strength can be compared only within the same leaf.

­
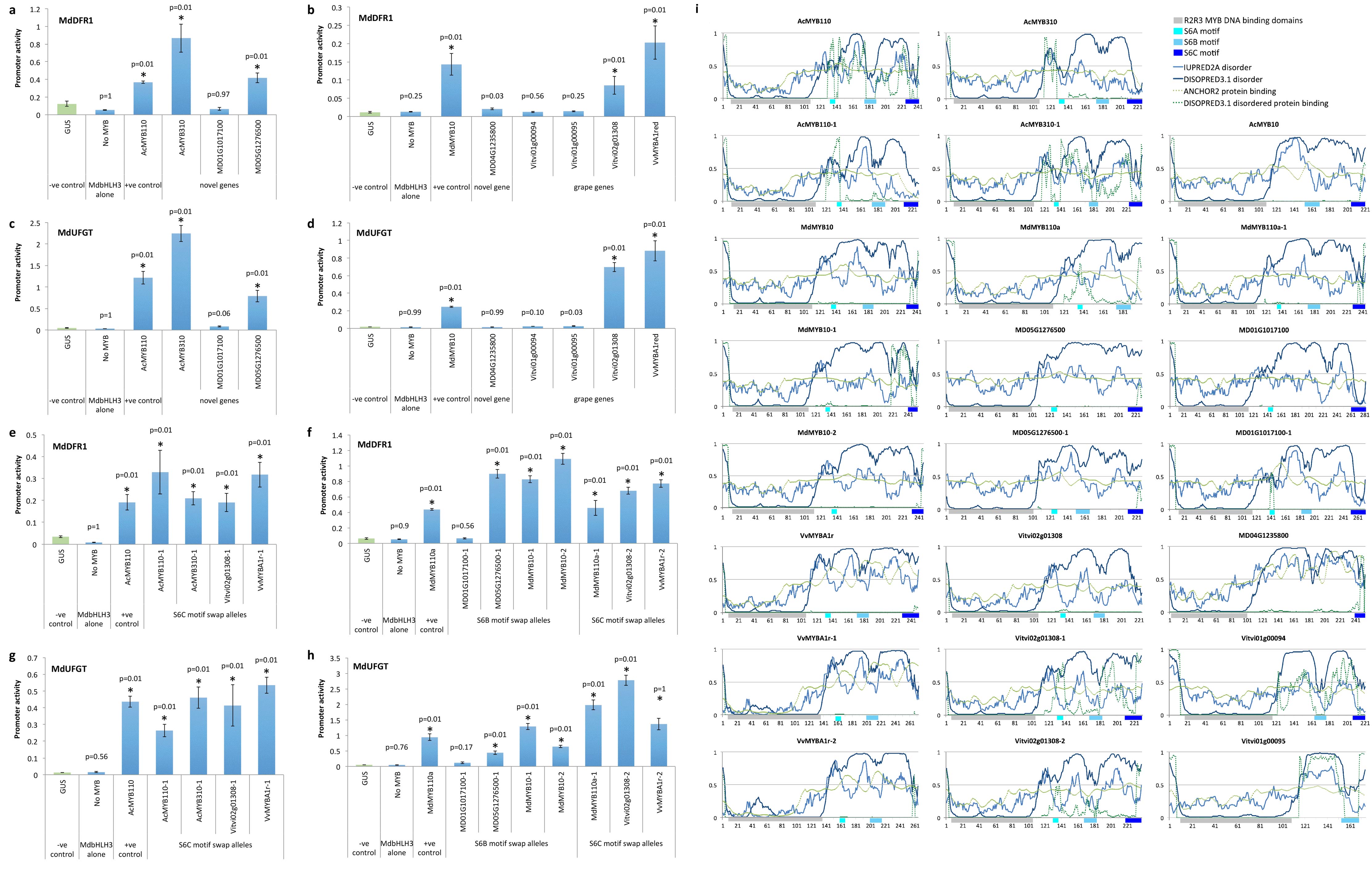


**Figure S5. Assessment of MYB proteins and their modified alleles by dual-luciferase assay and protein structure prediction. a-h** Dual-luciferase assay assessed whether MYB genes or their modified alleles activated promoters of late-stage anthocyanin genes apple ***dihydroflavonol-4-reductase 1*** (*MdDFR1*) (**a,b,e,f**) and **apple *UDP-glucose:flavonoid 3-O-glycosyltransferase 1* (***MdUFGT1*) (**c,d,g,h**). The assay involved transient expression of MYBs and dual-luciferase promoter constructs in leaves of *Nicotiana benthamiana* with MdbHLH3 as a co-factor in addition to endogenous tobacco basic helix-loop-helix (bHLH) transcription factors. Results from different experimental runs are presented in different graphs. Asterisks indicate significant promoter activation (p=0.01, Wilcoxon one-sided rank sum test) when compared with the negative control β-glucuronidase (GUS). Error bars indicate the SEM for four replicates. **i** Disorder and protein binding scores along protein sequences of natural and modified alleles of subgroup 6 R2R3 MYBs were predicted using IUPred2A ^46^ and DISOPRED3 ^47^ algorithms. Amino acid position is plotted along the x-axis, with conserved MYB DNA binding domains and C-terminal motifs denoted by filled grey and blue boxes. Clear boxes outlined in blue highlight regions that align with the sequence motifs of other MYBs but are considerably diverged from the consensus. Predicted disordered regions are defined by a disorder score of 0.5 to 1, while protein binding regions are defined by a binding score of 0.5 to 1. Note that, unlike ANCHOR2, DISOPRED3.1 specialises in predicting protein binding probability for disordered regions and does not make predictions for ordered regions.

**Figure S6. Codon alignment of select grape (VvMYB), tomato (SlAN), *Arabidopsis* (AtMYB), apple (MdMYB), and kiwifruit (AcMYB) genes that represent the sequence diversity of eudicot subgroup 6 R2R3 MYB genes, including both genes that activate anthocyanin and those that do not.** Codon nucleotide alignment bases that are identical to the consensus nucleotide are printed in grey without highlighting, while those that differ are printed in black and highlighted in either red (adenine), green (thymine), yellow (guanine), or blue (cytosine). Protein translations just below the corresponding nucleotide sequence are likewise printed in grey without highlighting, while those that differ are printed in black and highlighted in amino acid-specific colours. Identity is plotted for individual nucleotide positions with green bars indicating 100% identity, yellow bars indicating identity ≥ 30% but <100 and red bars indicating identity < 30%. The dendrogram on the left depicts sequence similarity deduced by the neighbour-joining method. Tandem R2R3 domains are annotated in grey and conserved motifs are in blue, with the previously described subgroup 6 motif ^16^ annotated as ‘S6A’ and two novel motifs annotated as ‘S6B’ and ‘S6C’. (provided as a separate file)

**Supplementary datasheets**

**Dataset S1 (sheet in Excel workbook). Relaxed PHYLIP codon alignment of informative R2R3 positions of 846 functional R2R3 and 3R MYBs of *Arabidopsis*, apple, grape, kiwifruit and tomato used in phylogenetic reconstruction with iqtree software.**

**Dataset S2 (sheet in Excel workbook). Relaxed PHYLIP codon alignment of informative R2R3 positions of 791 functional and non-functional R3, R2R3 and 3R MYBs of *Arabidopsis*, apple, grape, and kiwifruit used in phylogenetic reconstruction with RAxML software.**

**Dataset S3 (sheet in Excel workbook). Genomic location, exon-intron structure, subgroup and R2R3 sequence of R3, R2R3 and 3R MYBs of grape, kiwifruit and apple identified in this study.** Also included are genomic location, subgroup and R2R3 sequence of *Arabidopsis* and tomato MYBs identified in this study.

**Supplementary Materials and Methods**

**Genomic analysis**

High-quality paired-end RNA-sequencing datasets of 75 to 150 bp read length, spanning cultivars and tissue types (Table S1), were mapped to reference genomes using STAR aligner ^1^ and *de novo* assembled into transcripts using Trinity ^2^ and rnaSPADES ^3^. *De novo* transcripts were mapped onto the genome with GMAP ^4^ and the resulting annotations refined and combined with publicly available annotations using PASA ^5^. Commands are included in the nextflow pipeline automating this process (Code S1). RNA-seq read coverage between *de novo* transcripts and the gene annotations they produced was compared by mapping to transcripts with Bowtie 2 ^6^.

**Generation of MYB constructs**

Linear Golden Gate-compatible fragments of pUC57-Kan plasmids (Genewiz® LLC, genewiz.com) containing either MYB constructs or the motif regions to be swapped were generated by iProof™ PCR (BioRad Laboratories, Inc., California, USA) using 10 μL of 5x HF reaction buffer, 1 μL of 10 mM each dNTPs, 5 μL of 5 μM each forward and reverse primers containing BsaI sites (Table S2), 0.5 μL iProof enzyme, 10 ng template and autoclaved Milli-Q® water (MilliporeSigma, Massachusetts, USA) to 50 μL. PCR was initiated by 1 minute of denaturation at 98**°**C, followed by 30 cycles of 10 seconds of denaturation at 98**°**C, 10 seconds annealing at the temperature recommended for iProof, and extension at 72**°**C for 15 to 30 s per kb. A final 10-minute extension was performed at 72**°**C. Motif-spanning regions swapped between MYBs ranged from 14 to 58 amino acids. For Golden Gate assembly, equimolar amounts of PCR products purified using the Zymo DNA Clean and Concentrator™-5 kit (Zymo Research, Irvine, California, USA) were combined to a total of 100 ng and added to a 20-μL reaction of 1x T4 DNA ligase buffer (Promega™ Corporation, Wisconsin, USA) containing 1 μL of BsaI-HFv2 (New England BioLabs® Inc., Massachusetts, USA), and 1 μL of T4 DNA ligase (Promega Corporation, Wisconsin, USA). Reactions were incubated for 25 cycles alternating between 37**°**C for 90 seconds and 16**°**C for 3 minutes, followed by 5 minutes at 37**°**C and a final 10 minutes at 80**°**C. Then 2 μL of assembly reaction was immediately transformed into 20 to 50 μL of competent *Escherichia coli* DH5α cells by 45-second heat shock at 42**°**C and subsequent recovery in 0.5 to 1 mL of SOC medium. Final expression plasmids were produced using the LR Clonase II™ kit for Gateway™ recombination (Thermo Fisher Scientific, Massachusetts, USA). In short, 150 ng of the destination vector pHEX2 was combined with 50 ng of the entry clone plasmid and 2 μL of LR clonase II™ enzyme in a 10-μL reaction in either water or Tris-EDTA (TE) buffer at pH 8.0. Reactions were incubated at 25**°**C for 1 hour before treatment with 1 μL of the provided Proteinase K solution at 37**°**C for 10 minutes. Then 1 μL of the reaction was transformed into 20 to 50 μL of competent *E. coli* DH5α cells as described. Transformants were selected on LB agar plates with the appropriate antibiotic (100 μg/mL spectinomycin for pHEX2 and 50 μg/mL kanamycin for pUC-Kan) and verified by Sanger sequencing (Macrogen, Inc., Seoul, Korea). Plasmids for subsequent PCR and transformation of *Agrobacterium tumefaciens* were miniprepped using the Zyppy™ Plasmid Miniprep kit (Zymo Research, Irvine, California, USA) according to the manufacturer’s instructions for 3 mL overnight liquid culture of *E. coli* transformants in LB.

**Dual-luciferase assay**

MYB constructs driven by the *35S* promoter on pHEX2 were individually electrotransformed into 20 to 50 μL of *Agrobacterium tumefaciens* GV3101, with transformants recovered in 1 mL of SOC and selected on LB plates containing rifampicin 15 μg/mL, gentamycin 10 μg/mL, and spectinomycin 50 μg/mL. Transformed *Agrobacterium tumefaciens* GV3101 carrying a construct either for apple MdbHLH3 overexpression under the *35S* promoter, β-glucuronidase (GUS) overexpression under the *35S* promoter, dual-luciferase expression including the apple ***dihydroflavonol-4-reductase 1*** (*MdDFR1)* promoter, or dual-luciferase expression including the **apple *UDP-glucose:flavonoid 3-O-glycosyltransferase 1* (***MdUFGT1)* promoter were generated previously. For the assay, glycerol stocks of *Agrobacterium tumefaciens* were streaked onto selective LB agar plates and incubated at 28**°**C, followed by restreaking onto a fresh selective plate, overnight incubation at 28**°**C, and suspension in 5 mL of infiltration buffer (10 mM MgCl_2_, 10 μM acetosyringone) to an OD_600_ between 1 to 2. The suspensions were incubated at room temperature for 2 hours with gentle shaking. OD_600_ was then adjusted to between 0.8 and 1, and each *MYB* suspension was combined with the *MdbHLH3* suspension and one of the dual-luciferase suspensions in a 9:9:2 ratio. This final mixture was inoculated on either side of the midvein on the abaxial surface of 4-week-old *Nicotiana benthamiana* leaves using a needleless 1-mL syringe. A mixture of *GUS* and dual-luciferase suspension in a 9:1 ratio was inoculated as control, with an additional mixture of *MdbHLH3* and dual-luciferase suspension in a 9:1 ratio serving as a second control. Four days after inoculation ^7^, *MdDFR1* and *MdUFGT1* promoter activation of luciferase expression was compared with *35S* promoter-driven renilla expression by transferring 3-mm diameter leaf punches from infiltrated patches into a white 96-well luminescence plate. Leaf punches were manually ground in 50 μL of phosphate buffered saline (PBS) solution, and chemiluminiscence quantified with the DLAR-2 dual-luciferase assay reagent kit (Targeting Systems, California, USA). For the assay, 50 μL of luciferase substrate was added to each well, followed by 10 seconds of shaking and 10 minutes of room temperature incubation prior to signal quantification. Next, 50 μL of renilla substrate was added to the well and processed as for luciferase substrate. Activation of *MdDFR1* and *MdUFGT1* promoters was standardised among leaves by calculating the ratio between luciferase and renilla signals. From at least two independent experiments, the four replicates with the highest renilla activity, exceeding 1300 units, were used to statistically evaluate (Wilcoxon one-sided rank sum test, p=0.01) whether activation of *MdDFR1* and *MdUFGT1* promoters driven by MYB constructs exceeded that driven non-specifically by the negative control protein β-glucuronidase (GUS).

**Supplementary Tables**

**Table S1. RNA-sequencing data sets used for identification of novel MYBs and improvement of gene annotations.** Abbreviation: PFR = The New Zealand Institute for Plant and Food Research.

| **Species and**  **Cultivar/Variety** | | **Tissue(s)** | **Data location** | **Data access source** | **Associated publication** |
| --- | --- | --- | --- | --- | --- |
| *Actinidia chinensis* | ‘Zesy002’ | Stem, upper leaf, shoot tips across seasons, axillary buds across seasons | PFR | Erika Varkonyi-Gasic, Charlotte Voogd, Niels Nieuwenhuizen, Erik Rikkerink, Joel Vanneste | N/A |
|  | ‘Hort16A’ | Fruit flesh and peel | PFR | Kathy Schwinn, Niels Nieuwenhuizen | N/A |
|  | ‘Hort22D’ | Fruit flesh and peel | PFR | Kathy Schwinn | N/A |
|  | ‘Zes006’ | Fruit flesh | PFR | Simona Nardozza | ^8^ |
| *Malus*  x *domestica* | ‘Golden Delicious’ | Fruit peel, leaf scars, bud scars | PFR | Charles Dwamena, Kui Lin-Wang, Joanna Bowen, Brogan McGreal | In preparation |
|  | ‘Granny Smith’ | Fruit peel | PFR | Charles Dwamena | N/A |
|  | ‘Granny Smith’ x ‘Royal Gala’ | Fruit peel | PFR | Charles Dwamena | N/A |
|  | ‘Royal Gala’ | Fruit flesh and peel, leaf, leaf scars, bud scars | PFR | Charles Dwamena, Joanna Bowen, Brogan McGreal | In preparation |
|  | Host (5)^9^ | Leaf | PFR | Joanna Bowen | In preparation |
|  | Host (8)^9^ | Leaf | PFR | Joanna Bowen | In preparation |
| *Vitis vinifera* | ‘Jingxiangyu’ | Leaf | Sequence read archive SRP091989 | https://www.ncbi.nlm.nih.gov/sra?term=SRP091989 | ^10^ |
|  | Cabernet Sauvignon | Wood | European nucleotide archive ERP012789 | https://www.ebi.ac.uk/ena/data/view/PRJEB11405 | N/A |
|  | ‘Nebbiolo’ | Fruit, leaf, bud, stem | Sequence read archive SRP108201 | https://www.ncbi.nlm.nih.gov/sra?term=SRP108201 | ^11^ |
|  | Pinot noir | Fruit peel | PFR | Rebecca Henry-Kirk, Jeff Bennett | N/A |
|  | Sauvignon blanc | Fruit peel and flesh | PFR | Rebecca Henry-Kirk, Jeff Bennett | N/A |
|  | Tempranillo | Leaf | Sequence read archive SRP067967 | https://www.ncbi.nlm.nih.gov/sra?term=SRP067967 | ^12^ |

**Table S2. Primers used in Golden Gate cloning of modified subgroup 6 alleles of apple, grape and kiwifruit, where overhangs for Golden Gate cloning are highlighted in bold.** Abbreviations: Ext. time = Extension time for elongation step in PCR cycle; s = seconds; min = minute.

| **Construct** | **Forward primer (name: sequence)** | **Reverse primer (name: sequence)** | **Template** | **Product size (bp)** | **Ta (°C)** | **Ext. time** |
| --- | --- | --- | --- | --- | --- | --- |
| VvMYBA1r-1 (small S6C swap from Vitvi02g01308) | JAR021: ATACAGGTCTCA**TCCT**TTTGAA ATGGGCCTCATTG | JAR008: ACTAGTAGGTCTCG**CAGA**GCG  CAGATACCAAATAC | pUC57-Kan Vitvi02g01308 | 1069 | 62.7 | 20 s |
|  | JAR009: AATAATAGGTCTCG**TCTG**CTGA  AGCCAGTTACC | JAR022: TATAATAGGTCTCG**AGGA**AAA  TCACCCTCACCTC | pUC57-Kan VvMYBA1r | 2641 | 55.2 | 40 s |
| VvMYBA1r-2 (large S6C swap from Vitvi02g01308) | JAR023: ACAGCTGGTCTCG**GCAT**CAGCT  GTAGATACCAATGG | JAR008: ACTAGTAGGTCTCG**CAGA**GCG  CAGATACCAAATAC | pUC57-Kan Vitvi02g01308 | 1110 | 62.8 | 20 s |
|  | JAR009: AATAATAGGTCTCG**TCTG**CTGA  AGCCAGTTACC | JAR024: ACAGCTGGTCTCG**ATGC**AGTTT  CTTCTGTCCTGAG | pUC57-Kan VvMYBA1r | 2576 | 57.1 | 40 s |
| Vitvi02g01308-1 (small S6C swap from VvMYBA1r) | JAR007: ATCTCAGGTCTCT**TCCA**TTTGAT GTGGGCTTCTGG | JAR008: ACTAGTAGGTCTCG**CAGA**GCG  CAGATACCAAATAC | pUC57-Kan VvMYBA1r | 1088 | 62.8 | 20 s |
|  | JAR009: AATAATAGGTCTCG**TCTG**CTGA  AGCCAGTTACC | JAR010: CAACAGGTCTCC**TGGA**AAATC AAAATTCCCACCTC | pUC57-Kan Vitvi02g01308 | 2494 | 56.3 | 40 s |
| Vitvi02g01308-2 (large S6C swap from VvMYBA1r) | JAR025: CATACAGGTCTCA**GCAA**CACA GAAAAAGGGACCCATG | JAR008: ACTAGTAGGTCTCG**CAGA**GCG  CAGATACCAAATAC | pUC57-Kan VvMYBA1r | 1152 | 62.8 | 20 s |
|  | JAR009: AATAATAGGTCTCG**TCTG**CTGA  AGCCAGTTACC | JAR026: TCATACAGGTCTCA**TTGC**AGTT  TCGTCAGCCAG | pUC57-Kan Vitvi02g01308 | 2453 | 57.1 | 40 s |
| MdMYB110a-1 (S6C addition from MdMYB10) | JAR027: CTATCAGGTCTCG**AGGA**TACTT TTGAAAGAGCTGC | JAR008: ACTAGTAGGTCTCG**CAGA**GCG  CAGATACCAAATAC | pUC57-Kan MdMYB10 | 1204 | 59.7 | 20 s |
|  | JAR009: AATAATAGGTCTCG**TCTG**CTGA  AGCCAGTTACC | JAR028: TCATACAGGTCTCA**TCCT**CGTC  TTCTAACAAGGTCTTC | pUC57-Kan MdMYB110a | 2416 | 55.0 | 40 s |
| MD01G1017100-1 (S6B swap from MdMYB10) | JAR034: GTATACAGGTCTCA**TACG**CCAC CACAAACG | JAR035: GTATACAGGTCTCA**TGCT**GGG ATATGCAGCTC | pUC57-Kan MdMYB10 | 127 | 54.4 | 5 s |
|  | JAR036: GTATACAGGTCTCA**AGCA**GTTT GCAGTTAGAGGATCTC | JAR037: GTATACAGGTCTCA**CGTA**TGAT TATTATGTTGTTGTATATTAGAATTAG | pUC57-Kan MD01G1017100 | 3570 | 56.4 | 1 min |
| **Construct** | **Forward primer (name: sequence)** | **Reverse primer (name: sequence)** | **Template** | **Product size (bp)** | **Ta (°C)** | **Ext. time** |
| MD05G1276500-1 (S6B swap from MdMYB10) | JAR034: GTATACAGGTCTCA**TACG**CCAC CACAAACG | JAR035: GTATACAGGTCTCA**TGCT**GGG ATATGCAGCTC | pUC57-Kan MdMYB10 | 127 | 54.4 | 5 s |
|  | JAR038: GTATACAGGTCTCA**AGCA**TAG GGTTAGGGGAAGAC | JAR039: GTATACAGGTCTCA**CGTA**CTAC AGTTCTCTTCAGATTT | pUC57-Kan MD05G1276500 | 3438 | 52.8 | 1 min |
| MdMYB10-1 (S6B swap from MD01G1017100) | JAR040: GTATACAGGTCTCA**AGCA**TTGA GTTAGAGGAAG | JAR041: GTATACAGGTCTCA**CGTA**CTTA AATCTTCTGCTG | pUC57-Kan MdMYB10 | 3486 | 51.6 | 1 min |
|  | JAR042: GTATACAGGTCTCA**TACG**TTAT TACAAGAGCCATTGG | JAR043: GTATACAGGTCTCA**TGCT**ACAT AATAGTGCTCTTTCAAC | pUC57-Kan MD01G1017100 | 160 | 53.0 | 5 s |
| MdMYB10-2 (S6B swap from MD05G1276500) | JAR040: GTATACAGGTCTCA**AGCA**TTGA GTTAGAGGAAG | JAR041: GTATACAGGTCTCA**CGTA**CTTA AATCTTCTGCTG | pUC57-Kan MdMYB10 | 3486 | 51.6 | 1 min |
|  | JAR044: GTATACAGGTCTCA**TACG**TTAT TACAGACATCATCACCACCTACAG | JAR045: GTATACAGGTCTCT**TGCT**AGTA AATGGTGTTCCATCAATATTGTCTGTATCATG | pUC57-Kan MD05G1276500 | 130 | 63.6 | 5 s |
| AcMYB110-1 (S6C swap from AcMYB310) | JAR032: GTATACAGGTCTCA**GACT**TTGG  GGTAATATTGTCATCG | JAR008: ACTAGTAGGTCTCG**CAGA**GCG  CAGATACCAAATAC | pUC57-Kan AcMYB310 | 1091 | 60.7 | 20 s |
|  | JAR009: AATAATAGGTCTCG**TCTG**CTGA AGCCAGTTACC | JAR033: AGAATCGGTCTCC**AGTC**CTAAA ATTGGCTCCTCAAATATTGATC | pUC57-Kan AcMYB110 | 2482 | 57.1 | 40 s |
| AcMYB310-1 (S6C swap from AcMYB110) | JAR030: GTATACAGGTCTCA**GACT**CCAA TCGGCTGGTGAC | JAR008: ACTAGTAGGTCTCG**CAGA**GCG  CAGATACCAAATAC | pUC57-Kan AcMYB110 | 1130 | 59.4 | 20 s |
|  | JAR009: AATAATAGGTCTCG**TCTG**CTGA  AGCCAGTTACC | JAR031: GTATACAGGTCTCA**AGTC**CGG  CAATGGGTAGC | pUC57-Kan AcMYB310 | 2479 | 57.1 | 40 s |

**Table S3. Nucleotide and protein sequences of modified subgroup 6 alleles that explore the effect of sequence variation at S6B and S6C motifs in apple, grape and kiwifruit.** Regions swapped between strong or weak alleles and vice versa are highlighted in bold. Abbreviations: Nt = nucleotide, Pr = protein.

| VvMYBA1r-1 (small S6C swap from Vitvi02g01308) | Nt | ATGGAGAGCTTAGGAGTTAGAAAGGGTGCATGGATCCAAGAAGAGGATGTTCTCCTGAGGAAATGCATTGAGAAATATGGAGAAGGAAAGTGGCATCTGGTTCCCCTCCGAGCAGGGTTGAATAGATGCCGAAAAAGCTGCAGGTTGAGATGGCTCAATTATTTGAAGCCGGATATCAAGAGAGGAGAGTTTGCATTAGACGAGGTTGATCTCATGATTAGGCTTCACAATTTGTTGGGGAACAGATGGTCCTTGATTGCGGGTAGGCTTCCAGGGAGGACTGCTAATGATGTCAAGAACTATTGGCATAGTCACCACTTCAAAAAGGAGGTTCAGTTCCAGGAAGAAGGGAGAGATAAACCCCAAACACATTCTAAAACCAAAGCTATAAAGCCTCACCCTCACAAGTTCTCCAAAGCCTTGCCAAGGTTTGAACTAAAAACTACAGCTGTGGATACTTTTGACACACAAGTCAGTACTTCCAGTAAGCCATCATCCACGTCACCACAACCGAATGATGACATCATATGGTGGGAAAGCCTGTTAGCTGAGCATGCTCCAATGGATCAAGAAACTGACTTTTCGGCTTCTGGAGAGATGCTTATCGCAAGCCTCAGGACAGAAGAAACTGCAACACAGAAAAAGGGACCCATGGATGGTATGATTGAACAAATCCAGGGAGGTGAGGGTGATTTTCC**TTTTGAAATGGGCCTCATTGGAGAAGCCATGATATAA** |
| --- | --- | --- |
|  | Pr | MESLGVRKGAWIQEEDVLLRKCIEKYGEGKWHLVPLRAGLNRCRKSCRLRWLNYLKPDIKRGEFALDEVDLMIRLHNLLGNRWSLIAGRLPGRTANDVKNYWHSHHFKKEVQFQEEGRDKPQTHSKTKAIKPHPHKFSKALPRFELKTTAVDTFDTQVSTSSKPSSTSPQPNDDIIWWESLLAEHAPMDQETDFSASGEMLIASLRTEETATQKKGPMDGMIEQIQGGEGDFP**FEMGLIGEAMI** |
| VvMYBA1r-2 (large S6C swap from Vitvi02g01308) | Nt | ATGGAGAGCTTAGGAGTTAGAAAGGGTGCATGGATCCAAGAAGAGGATGTTCTCCTGAGGAAATGCATTGAGAAATATGGAGAAGGAAAGTGGCATCTGGTTCCCCTCCGAGCAGGGTTGAATAGATGCCGAAAAAGCTGCAGGTTGAGATGGCTCAATTATTTGAAGCCGGATATCAAGAGAGGAGAGTTTGCATTAGACGAGGTTGATCTCATGATTAGGCTTCACAATTTGTTGGGGAACAGATGGTCCTTGATTGCGGGTAGGCTTCCAGGGAGGACTGCTAATGATGTCAAGAACTATTGGCATAGTCACCACTTCAAAAAGGAGGTTCAGTTCCAGGAAGAAGGGAGAGATAAACCCCAAACACATTCTAAAACCAAAGCTATAAAGCCTCACCCTCACAAGTTCTCCAAAGCCTTGCCAAGGTTTGAACTAAAAACTACAGCTGTGGATACTTTTGACACACAAGTCAGTACTTCCAGTAAGCCATCATCCACGTCACCACAACCGAATGATGACATCATATGGTGGGAAAGCCTGTTAGCTGAGCATGCTCCAATGGATCAAGAAACTGACTTTTCGGCTTCTGGAGAGATGCTTATCGCAAGCCTCAGGACAGAAGAAACTGCA**TCAGCTGTAGATACCAATGGAGGTGGGAATTTTGATTTTCCTTTTGAAATGGGCCTCATTGGAGAAGCCATGATATAA** |
|  | Pr | MESLGVRKGAWIQEEDVLLRKCIEKYGEGKWHLVPLRAGLNRCRKSCRLRWLNYLKPDIKRGEFALDEVDLMIRLHNLLGNRWSLIAGRLPGRTANDVKNYWHSHHFKKEVQFQEEGRDKPQTHSKTKAIKPHPHKFSKALPRFELKTTAVDTFDTQVSTSSKPSSTSPQPNDDIIWWESLLAEHAPMDQETDFSASGEMLIASLRTEETA**SAVDTNGGGNFDFPFEMGLIGEAMI** |
| Vitvi02g01308-1 (small S6C swap from VvMYBA1r) | Nt | ATGGAGAGTTTGGGAGTTACAAAGGGAAAATGGACTCGGGAAGAAGATATTCTTTTAAGAAAATGTGTTCAGAAATATGGAGAAGAAAAGTGGCATCTGGTTCCACTCCGAGCAGGTTTGAGTAGATGCCGCAAAAGCTGTAGATTGAGATGGTTCAACTATTTAAAGCCAAATATTAAGAGAGGAAAATTTGCATCAGATGAAGTCGATCTCATGATTAGACTTCATAAGCTGTTGGGAAACAGATGGTCCTTGATTGCCGGCAGACTTCCAGGGAGGACTGCGAATGATGTCAAGAATTACTGGCACCATCATCGCTTCAAGAAGATGGTTCCCATCCAGGAAAAAGGGAAAGATAAAGCCCAAACCAATTCTGAAAACACTCTGATAAAGCCTAAGCCTTGCAAACTCTTTCTAGGGTTCACATTCAAAACTACAGCTGTGGATGCTTCTGAGACACAAGGCAGTACTTGTAGTGAGCTGCAACCCACACTGGTCCAACCAAACCAGGATATTTTATGGTGGGAAAGCCTATTCGCTGACCATACCATTGAAGATCAGGAGCTTATTGCCAGCCTCCTGGCTGACGAAACTGCATCAGCTGTAGATACCAATGGAGGTGGGAATTTTGATTTTCC**ATTTGATGTGGGCTTCTGGGATACACCCAACACACAAGTAAATCATTTGATCTGA** |
|  | Pr | MESLGVTKGKWTREEDILLRKCVQKYGEEKWHLVPLRAGLSRCRKSCRLRWFNYLKPNIKRGKFASDEVDLMIRLHKLLGNRWSLIAGRLPGRTANDVKNYWHHHRFKKMVPIQEKGKDKAQTNSENTLIKPKPCKLFLGFTFKTTAVDASETQGSTCSELQPTLVQPNQDILWWESLFADHTIEDQELIASLLADETASAVDTNGGGNFDFPF**DVGFWDTPNTQVNHLI** |
| Vitvi02g01308-2 (large S6C swap from VvMYBA1r) | Nt | ATGGAGAGTTTGGGAGTTACAAAGGGAAAATGGACTCGGGAAGAAGATATTCTTTTAAGAAAATGTGTTCAGAAATATGGAGAAGAAAAGTGGCATCTGGTTCCACTCCGAGCAGGTTTGAGTAGATGCCGCAAAAGCTGTAGATTGAGATGGTTCAACTATTTAAAGCCAAATATTAAGAGAGGAAAATTTGCATCAGATGAAGTCGATCTCATGATTAGACTTCATAAGCTGTTGGGAAACAGATGGTCCTTGATTGCCGGCAGACTTCCAGGGAGGACTGCGAATGATGTCAAGAATTACTGGCACCATCATCGCTTCAAGAAGATGGTTCCCATCCAGGAAAAAGGGAAAGATAAAGCCCAAACCAATTCTGAAAACACTCTGATAAAGCCTAAGCCTTGCAAACTCTTTCTAGGGTTCACATTCAAAACTACAGCTGTGGATGCTTCTGAGACACAAGGCAGTACTTGTAGTGAGCTGCAACCCACACTGGTCCAACCAAACCAGGATATTTTATGGTGGGAAAGCCTATTCGCTGACCATACCATTGAAGATCAGGAGCTTATTGCCAGCCTCCTGGCTGACGAAACTGCA**ACACAGAAAAAGGGACCCATGGATGGTATGATTGAACAAATCCAGGGAGGTGAGGGTGATTTTCCATTTGATGTGGGCTTCTGGGATACACCCAACACACAAGTAAATCATTTGATCTGA** |
|  | Pr | MESLGVTKGKWTREEDILLRKCVQKYGEEKWHLVPLRAGLSRCRKSCRLRWFNYLKPNIKRGKFASDEVDLMIRLHKLLGNRWSLIAGRLPGRTANDVKNYWHHHRFKKMVPIQEKGKDKAQTNSENTLIKPKPCKLFLGFTFKTTAVDASETQGSTCSELQPTLVQPNQDILWWESLFADHTIEDQELIASLLADETA**TQKKGPMDGMIEQIQGGEGDFPFDVGFWDTPNTQVNHLI** |
| MdMYB110a-1 (S6C addition from MdMYB10) | Nt | ATGGAGGGATGTAACGTTAACTTGAGTGTGATGAGAAAAGGTGCCTGGACTCGAGAGGAAGATGATCTTCTCAGGCAGTGCATTGAGATTCTTGGAGAAGGAAAGTGGCACCAAGTTCCATACAAAGCAGGCTTAAACAGGTGCAGGAAGAGCTGCAGACTAAGATGGTTGAACTATCTGAAGCCAAATATCAAGAGAGGAGACTTTACTGAGGATGAAGTAGATCTAATAATTAGGCTTCACAAGCTTTTAGGAAACAGGTGGTCGTTGATTGCTGGAAGACTTCCAGGAAGAACAGCGAATGATGTGAAAAATTATTGGAACACTCGATTACGGATCAATTCTCGCATGAAAACATTGCAAAATAATTCCCAAGAAACAAGAAAGACCATTGTGATCAGACCTCAACCCCGAAGTTTCATAAAAAGTTCAAATTACTTGAGCAGTAAAGAACCAATTATAGACCATATTCAATCAGAAGAGGATTTAAGTACGTCACCACAAACGTCGTCGTCGACAAACAATGGAAATGATTGGTGGAAGACCTTGTTAGAAGACG**AGGATACTTTTGAAAGAGCTGCATATCCCAGCATTGAGTTAGAGGAAGAACTCTTCACAAGTTTTTGGTTTGATGATCGACTGTCGCCAAGATCATGCGCCAATTTTCCTGAAGGACAAAGTAGAAGTGAATTCTCCTTTAGCACGGACCTTTGGAATCATTCAAAAGAAGAATAG** |
|  | Pr | MEGCNVNLSVMRKGAWTREEDDLLRQCIEILGEGKWHQVPYKAGLNRCRKSCRLRWLNYLKPNIKRGDFTEDEVDLIIRLHKLLGNRWSLIAGRLPGRTANDVKNYWNTRLRINSRMKTLQNNSQETRKTIVIRPQPRSFIKSSNYLSSKEPIIDHIQSEEDLSTSPQTSSSTNNGNDWWKTLLED**EDTFERAAYPSIELEEELFTSFWFDDRLSPRSCANFPEGQSRSEFSFSTDLWNHSKEE** |
| MD01G1017100-1 (S6B swap from MdMYB10) | Nt | ATGGAAGAGGGTAATTCGTTGGGAGTAGTGAGAAAAGGTGCATGGACTAAAGAGGAAGACGATCTTCTCCAGCACTTCGTTCAACAGCATGGTGAAGGAAAATGGCGTCAGGTTCCTCTCAAAGCAGGTTTAAACAGATGCAGGAAAAGCTGCAGACTGAGGTGGTTGAACTACTTGAAGCCAAGTATCAAAAGAGGAGACTTCGGAGAGGATGAAATAGATCTAATGGTCAGACTTCACAAACTAGTAGGAAACAGGTGGTCAATGATTGCCGGAAGACTTCCAGGAAGAACAGCAAACGATGTGAAAAATTACTGGAGTACTCGATTAAGGTGGAAGAAGTCGGCTTCTGGTGACAAGTTGAAAAATCATAAACCCCTACAACAAGTAGCTAAAGTAGAAACAACAAAGACCGTCGTAATACGACCTCGACCACGAACCTTCTCCAAAAACTTGAATTATTTGAGCCGCAAATTAGCTGCAACTAATTCTAATATACAACAACATAATAATCA**TACGCCACCACAAACGTCGTCGTCAACAAAGAATGGAAATGATTGGTGGGAGACCTTGTTAGAAGGTGAGGATACTTTTGAAAGAGCTGCATATCCCAGCA**GTTTGCAGTTAGAGGATCTCTTCACAAACTTTTGGATTGAAGAAGATGATGTTATGGCCCATACAACATCAACAACAAGAACAGGACTCAATTGTAGTATTACTGAACAGGAAGAAGAAGGTTTGATGAGTAGGAGTGACTACTTCTTCTCCTTTGATGACATGGATATTTGGAATAACTTAAGCATGTATTTGAACTAA |
|  | Pr | MEEGNSLGVVRKGAWTKEEDDLLQHFVQQHGEGKWRQVPLKAGLNRCRKSCRLRWLNYLKPSIKRGDFGEDEIDLMVRLHKLVGNRWSMIAGRLPGRTANDVKNYWSTRLRWKKSASGDKLKNHKPLQQVAKVETTKTVVIRPRPRTFSKNLNYLSRKLAATNSNIQQHNN**HTPPQTSSSTKNGNDWWETLLEGEDTFERAAYPSS**LQLEDLFTNFWIEEDDVMAHTTSTTRTGLNCSITEQEEEGLMSRSDYFFSFDDMDIWNNLSMYLN |
| MD05G1276500-1 (S6B swap from MdMYB10) | Nt | ATGAGGAAAGGTGCCTGGACTCAACAGGAAGATGATATTCTGAGGCAGTACGTTGAAAAGCATGGAGATGGAAAGTGGCACCAGGTTCCTCGCGAAACAGGTCTAAACAGATGCAGGAAAAGCTGCAGACTGAGGTGGTTGAACTATTTGAAGCCGAATCTCAAGAGCGGAGATTTCACAGAGGATGAAATAGATCTAATCCATAGACTTCAGAAACTTTTGGGAAACAGGTGGTCAATAATTGCTGGAAGACTCCCAGGAAGAACAGCAGGCAAGGTAAAAAATTATTGGAATAGCAAGCAACGAAAGGAGTTGGAATATATGAAGGATAAATCAAAAGAAAGAACAAAAGTCACATCCGTCATAAGACCTCAACCACGGAGGGCTAGAGTTGCAGTTTTAAAATCTGAAGAGAACTGTAG**TACGCCACCACAAACGTCGTCGTCAACAAAGAATGGAAATGATTGGTGGGAGACCTTGTTAGAAGGTGAGGATACTTTTGAAAGAGCTGCATATCCCAGCA**TAGGGTTAGGGGAAGACCTCTTCACAAACTTTTGGGTTGAAGATATTGCACAATCGACAACGGTAGGCATGAATTCTGCTGATGAAGGGTTACACATGAGTGGCAACTTTTCTTTTAGGGAGAACCTTTGGAATCTAGAAGAAGAGAGAACTAAGATTTAG |
|  | Pr | MRKGAWTQQEDDILRQYVEKHGDGKWHQVPRETGLNRCRKSCRLRWLNYLKPNLKSGDFTEDEIDLIHRLQKLLGNRWSIIAGRLPGRTAGKVKNYWNSKQRKELEYMKDKSKERTKVTSVIRPQPRRARVAVLKSEENCS**TPPQTSSSTKNGNDWWETLLEGEDTFERAAYPSI**GLGEDLFTNFWVEDIAQSTTVGMNSADEGLHMSGNFSFRENLWNLEEERTKI |
| MdMYB10-1 (S6B swap from MD01G1017100) | Nt | ATGGAGGGATATAACGAAAACCTGAGTGTGAGAAAAGGTGCCTGGACTCGAGAGGAAGACAATCTTCTCAGGCAGTGCGTTGAGATTCATGGAGAGGGAAAGTGGAACCAAGTTTCATACAAAGCAGGCCTAAACAGGTGCAGGAAGAGCTGCAGACAAAGATGGTTAAACTATCTGAAGCCAAATATCAAGAGAGGAGACTTTAAAGAGGATGAAGTAGATCTTATAATTAGACTTCACAGGCTTTTGGGAAACAGGTGGTCATTGATTGCTAGAAGACTTCCAGGAAGAACAGCAAATGCTGTGAAAAATTATTGGAACACTCGATTGCGGATCGATTCTCGCATGAAAACGGTGAAAAATAAATCTCAAGAAATGAGAGAGACCAATGTGATAAGACCTCAGCCCCAAAAATTCAACAGAAGTTCATATTACTTAAGCAGTAAAGAACCAATTCTAGACCATATTCAATCAGCAGAAGATTTAAG**TACGTTATTACAAGAGCCATTGGCAGTGACTTCATCATTAACACCTATAGAGATTAATGGAATTAGTGAAACCTTGTTAGGTGATGATAACAAAGACAGTAGTAGTACTGTTGAAAGAGCACTATTATGTAGCA**TTGAGTTAGAGGAAGAACTCTTCACAAGTTTTTGGTTTGATGATCGACTGTCGCCAAGATCATGCGCCAATTTTCCTGAAGGACAAAGTAGAAGTGAATTCTCCTTTAGCACGGACCTTTGGAATCATTCAAAAGAAGAATAG |
|  | Pr | MEGYNENLSVRKGAWTREEDNLLRQCVEIHGEGKWNQVSYKAGLNRCRKSCRQRWLNYLKPNIKRGDFKEDEVDLIIRLHRLLGNRWSLIARRLPGRTANAVKNYWNTRLRIDSRMKTVKNKSQEMRETNVIRPQPQKFNRSSYYLSSKEPILDHIQSAEDLS**TLLQEPLAVTSSLTPIEINGISETLLGDDNKDSSSTVERALLCS**IELEEELFTSFWFDDRLSPRSCANFPEGQSRSEFSFSTDLWNHSKEE |
| MdMYB10-2 (S6B swap from MD05G1276500) | Nt | ATGGAGGGATATAACGAAAACCTGAGTGTGAGAAAAGGTGCCTGGACTCGAGAGGAAGACAATCTTCTCAGGCAGTGCGTTGAGATTCATGGAGAGGGAAAGTGGAACCAAGTTTCATACAAAGCAGGCCTAAACAGGTGCAGGAAGAGCTGCAGACAAAGATGGTTAAACTATCTGAAGCCAAATATCAAGAGAGGAGACTTTAAAGAGGATGAAGTAGATCTTATAATTAGACTTCACAGGCTTTTGGGAAACAGGTGGTCATTGATTGCTAGAAGACTTCCAGGAAGAACAGCAAATGCTGTGAAAAATTATTGGAACACTCGATTGCGGATCGATTCTCGCATGAAAACGGTGAAAAATAAATCTCAAGAAATGAGAGAGACCAATGTGATAAGACCTCAGCCCCAAAAATTCAACAGAAGTTCATATTACTTAAGCAGTAAAGAACCAATTCTAGACCATATTCAATCAGCAGAAGATTTAAG**TACGTTATTACAGACATCATCACCACCTACAGAAAATGCTATTGATTCATGGAAGACCATGTTGCATGATACAGACAATATTGATGGAACACCATTTACTAGCA**TTGAGTTAGAGGAAGAACTCTTCACAAGTTTTTGGTTTGATGATCGACTGTCGCCAAGATCATGCGCCAATTTTCCTGAAGGACAAAGTAGAAGTGAATTCTCCTTTAGCACGGACCTTTGGAATCATTCAAAAGAAGAATAG |
|  | Pr | MEGYNENLSVRKGAWTREEDNLLRQCVEIHGEGKWNQVSYKAGLNRCRKSCRQRWLNYLKPNIKRGDFKEDEVDLIIRLHRLLGNRWSLIARRLPGRTANAVKNYWNTRLRIDSRMKTVKNKSQEMRETNVIRPQPQKFNRSSYYLSSKEPILDHIQSAEDLS**TLLQTSSPPTENAIDSWKTMLHDTDNIDGTPFTS**IELEEELFTSFWFDDRLSPRSCANFPEGQSRSEFSFSTDLWNHSKEE |
| AcMYB110-1 (S6C swap from AcMYB310) | Nt | ATGGAAACTGTTCCTTTAGGAGTGAGAAAGGGTGCATGGACTGAGGAAGAGGATAAGCTTCTCAAGAAGTGCATTGAGAAGTATGGAGAAGGAAAGTGGTACCAAATTCCTCTCAGAGCAGGATTGAACAGATGCAGAAAAAGCTGTAGATTGAGATGGTTAAACTATCTAAGGCCAAATATAAACAGAGGAATCTTTACAGCTGATGAAGTTGATCTCATTATAAGGCTTCATAAGCTGTTAGGCAACAGATGGTCGCTAATTGCGGGTCGACTTCCTGGACGAACATCAAATGATGTAAAAAATTATTGGAATACCCATCTCCAGAAGAAACTGATATCTACAAGAGAAGAGCCGATACCCAAAACCCAAAAGACGATCGTACCCAAAGTTACACGGCCTCAGCCTCGGATCTTCATGAAACACCAACCTTTGTTGGAGGGCAAAACTGTCATTGCAGATAGCATTCAACCAAGAGATGTTAACCTCCCCAAGCCATCCCCAACACCAACACCAACCAACAATGAAATATTGTGGTGGGACTACAAGATTCTGGGACCAGAAATTGACGATATGGGAATCAATTGGTCAATTGATGGATCAATATTTGAGGAGCCAATTTTAG**GACTTTGGGGTAATATTGTCATCGATGATGTGGAACTTTGGGATCTTAATAATTTTAGGTGA** |
|  | Pr | METVPLGVRKGAWTEEEDKLLKKCIEKYGEGKWYQIPLRAGLNRCRKSCRLRWLNYLRPNINRGIFTADEVDLIIRLHKLLGNRWSLIAGRLPGRTSNDVKNYWNTHLQKKLISTREEPIPKTQKTIVPKVTRPQPRIFMKHQPLLEGKTVIADSIQPRDVNLPKPSPTPTPTNNEILWWDYKILGPEIDDMGINWSIDGSIFEEPILG**LWGNIVIDDVELWDLNNFR** |
| AcMYB310-1 (S6C swap from AcMYB110) | Nt | ATGGATATCAAACCTTTAGGAGTAAGAAAAGGTGCATGGACACCAGAAGAAGATCATCTTCTCAAGACGTGTGTTGACAAGTATGGAGAAGGAAAGTGGCACCAAATTCCCCTCCAGGCAGGCTTAAATAGGTGCAGGAAAAGTTGTAGGCTTAGGTGGTTGAACTATTTGAAGCCAAATATAAAGAGAGGAAATTTTAGTGGTGACGAAATTGATTTGATGATCAGGCTTCATAAGCTTTTAGGCAACAGATGGTCATTAATTGCGGGTAGACTTCCGGGAAGAACGTCAAATGATGTTAAAAACTACTGGAACACTCATTTAAAAAAGAAGTCCATCGCATCTCAAAATACAAATCCTCAGATGTCCAAGGAGCAAAAAATTTTTCGGCCTCAACCTCGGGCCTTCTCGATAAATCTCCAATGGTTGCAGAGCAAAACTACGACTACAACTGCAGATCATATACAAACAAAGGACAACTTCGTTAATTCATCTCCTGTCTCAGCACTATGTGGTGATGGAACATCGTGGTGGGATGAAGTGGCAGTCGACCTTGAAATTAATGTTGGAAATAATTGCTGGTTCGAGGATGGCTCCACGGCGGAGCTACCCATTGCCGGACT**CCAATCGGCTGGTGACAGGCCTCTTCAACAAAATCAAAGGGATTGGAGTGATACAGTTATAGAAGATGTTGACCTATGGAATCTCTTAGGTGATGATTAA** |
|  | Pr | MDIKPLGVRKGAWTPEEDHLLKTCVDKYGEGKWHQIPLQAGLNRCRKSCRLRWLNYLKPNIKRGNFSGDEIDLMIRLHKLLGNRWSLIAGRLPGRTSNDVKNYWNTHLKKKSIASQNTNPQMSKEQKIFRPQPRAFSINLQWLQSKTTTTTADHIQTKDNFVNSSPVSALCGDGTSWWDEVAVDLEINVGNNCWFEDGSTAELPIAGL**QSAGDRPLQQNQRDWSDTVIEDVDLWNLLGDD** |

**References**

1 Dobin, A. *et al.* STAR: ultrafast universal RNA-seq aligner. *Bioinformatics* **29**, 15-21, doi:10.1093/bioinformatics/bts635 (2013).

2 Haas, B. J. *et al.* De novo transcript sequence reconstruction from RNA-seq using the Trinity platform for reference generation and analysis. *Nat Protoc* **8**, 1494-1512, doi:10.1038/nprot.2013.084 (2013).

3 Bushmanova, E., Antipov, D., Lapidus, A. & Prjibelski, A. D. rnaSPAdes: a de novo transcriptome assembler and its application to RNA-Seq data. *Gigascience* **8**, doi:10.1093/gigascience/giz100 (2019).

4 Wu, T. D. & Watanabe, C. K. GMAP: a genomic mapping and alignment program for mRNA and EST sequences. *Bioinformatics* **21**, 1859-1875, doi:10.1093/bioinformatics/bti310 (2005).

5 Haas, B. J. *et al.* Automated eukaryotic gene structure annotation using EVidenceModeler and the Program to Assemble Spliced Alignments. *Genome Biol* **9**, R7, doi:10.1186/gb-2008-9-1-r7 (2008).

6 Langmead, B. & Salzberg, S. L. Fast gapped-read alignment with Bowtie 2. *Nat Methods* **9**, 357-359, doi:10.1038/nmeth.1923 (2012).

7 Lotkowska, M. E. *et al.* The Arabidopsis Transcription Factor MYB112 Promotes Anthocyanin Formation during Salinity and under High Light Stress. *Plant Physiol* **169**, 1862-1880, doi:10.1104/pp.15.00605 (2015).

8 Nardozza, S. *et al.* Carbon starvation reduces carbohydrate and anthocyanin accumulation in red-fleshed fruit via trehalose 6-phosphate and MYB27. *Plant Cell Environ* **43**, 819-835, doi:10.1111/pce.13699 (2020).

9 Bus, V. G., Rikkerink, E. H., Caffier, V., Durel, C. E. & Plummer, K. M. Revision of the nomenclature of the differential host-pathogen interactions of Venturia inaequalis and Malus. *Annu Rev Phytopathol* **49**, 391-413, doi:10.1146/annurev-phyto-072910-095339 (2011).

10 Jiang, J. *et al.* Integrating Omics and Alternative Splicing Reveals Insights into Grape Response to High Temperature. *Plant Physiol* **173**, 1502-1518, doi:10.1104/pp.16.01305 (2017).

11 Gambino, G. *et al.* Whole-genome sequencing and SNV genotyping of 'Nebbiolo' (Vitis vinifera L.) clones. *Sci Rep* **7**, 17294, doi:10.1038/s41598-017-17405-y (2017).

12 Diaz-Riquelme, J. *et al.* Comparative genome-wide transcriptome analysis of Vitis vinifera responses to adapted and non-adapted strains of two-spotted spider mite, Tetranyhus urticae. *BMC Genomics* **17**, 74, doi:10.1186/s12864-016-2401-3 (2016).

13 Jaillon, O. *et al.* The grapevine genome sequence suggests ancestral hexaploidization in major angiosperm phyla. *Nature* **449**, 463-467, doi:10.1038/nature06148 (2007).

**Code S1. Nextflow pipeline “main.nf” file for improving genome annotations using RNA-sequencing data.** Critical steps in the pipeline are summarized in Figure 1.

#!/nextflow

// -----------------------------------------------------------------------

// README:

// This is a nextflow pipeline for automation of the several steps needed

// for gene annotation improvement using the PASA tool. This pipeline

// should be run in a directory where output sub-directories can be

// created.

//

// Below parameters for the pipeline must be provided in a config file,

// which may be named “nextflow.config”:

// 1. Paired-end reads in fastq format

// (Optional: Test sample with -profile sample)

// 2. Reference genome in fasta format

// 3. Annotation in gtf format

// 4. Sortmerna databases in fasta format and their index in db format

// 5. Group info for the reads files in groupinfo.csv (see nextflow.config)

// A sample config file is provided as the later part of this README

//

// The pipeline may be invoked in a linux environment with the command:

// “nextflow -C nextflow.config -log nextflow.log run main.nf

// -profile standard”

//

// One or more optional profiles can be chosen with -profile <profile1>...

// 1. standard (default, required): Contains basic parameters like fastq file

// location, command options, output directory, and so on

// 2. sample (optional): Tests the pipeline on a sample of the data

// 3. stopQCfail (optional): Does not halt the pipeline if QC check fails

//

// Example of nextflow run with optional profiles:

// ./nextflow run main.nf -profile standard,sample

// In the absence of an indicated profile, the standard profile is run

//

// ------------------------------------------------------------------------

// SAMPLE CONTENTS FOR A “nextflow.config” FILE (Note: One instance of the

// comment marker “//” will need to be removed if copying and pasting the

// below to create a config file):

//

// // Nextflow and reporting options

// nextflowVersion = '>=0.31.1' // Use version 0.31.1 or later

// timeline.enabled = true // Produce timeline.html

// report.enabled = true // Produce report.html, a workflow execution report

// dag.enabled = true // Produce a flowchart of the processes and channels

// dag.file = 'flowchart.pdf'

//

// // Profiles

// profiles{

//

// standard{

// params{

// // Reference genome and associated information

// annot= '/path/gene_models.gtf' // Path for the annotation file

// genome= '/path/genome.fasta'

// // Path for the reference genome file (fasta format)

// star.index= 'path/STARindex'

// // Path to genome indices generated by the STAR read aligner

// // If no index, set to ' ' for STAR --runMode genomeGenerate

//

// // Fastq input

// reads= 'path/*_R{1,2}[_.]*f*[qz]' // Path to fastq files

// // structure for paired read interpretation '_R{1,2}'

// cleanreads= 'path/*_R{1,2}.fastq.gz' // Only used with skipClean profile

// groupfile= "${baseDir}/groupinfo.csv"

// // Path to comma-separated file with header and data on

// // file pair ID and group correspondence, with file pair ID

// // defined as the file name up until _R1.fastq.gz (or similar)

//

// // Trimmomatic options

// Adapterfile='adapters.fasta'

// Pclip=':2:30:5' // Illuminaclip parameters

// SlidWindow=':4:15'

// // SlidingWindow: e.g 4 bases must have average quality score >= 15

// MinL=':75' // Drop reads below 75bp

// Leading=':10' // Clip bases at start if quality below 10

// Trailing=':10' // Clip bases at end if quality below 10

//

// // Sortmerna databases and their corresponding indices

// RRNADB='/path/sortmerna_rrna_db' // folder with databases

// SORTMERNADB="\

// ${RRNADB}/5s_sequences.fasta,${RRNADB}/5s_sequences:\

// ${RRNADB}/LSU_sequences.fasta,${RRNADB}/LSU_sequences:\

// ${RRNADB}/SSU_sequences.fasta,${RRNADB}/SSU_sequences"

//

// // STAR read aligner options

// star.ReadLen= 100 // Length of single read from paired-end

// star.IndexRAM= 120000000000 // value for --limitGenomeGenerateRAM

// star.IndexBinBits=18 // value for --genomeChrBinNbits

// star.IndexSjdbOver= star.ReadLen -1 // value for --sjdbOverhang

// star.IndexSjdbTrans= 'transcript_id'

// // value for --sjdbGTFtagExonParentTranscript

// star.IndexSjdbGene= 'gene_id' // value for --sjdbGTFtagExonParentGene

// star.SegmMin=0 // if 0, no chimeric output

// star.JunctMin=40 // Minimum overhang for a chimeric junction

// star.GapMax=0 // if 0, will be (2^winBinNbits)*winAnchorDistNbins

// star.IntronMax=0 // if 0, will be (2^winBinNbits)*winAnchorDistNbins

// star.outQSconvAdd=0 // For Illumina to Sanger conversion, set to -31

//

// // PASA options

// PASA.database= "${baseDir}/db.sqlite"

// // Name for SQLIte database to be generated by PASA

// PASA.options= """validate_alignments_in_db.dbi:--MIN_PERCENT_ALIGNED=90

validate_alignments_in_db.dbi:--MIN_AVG_PER_ID=95

// """ // multiline entry consisting of parameters for various PASA scripts

// PASA.maxIntron= 60000 // Maximum allowed intron size

//

// // Location of output directories

// outdirNF= "${baseDir}/nextflowlogs"

// outdir1= "${baseDir}/010.Trimmomatic"

// outdir2= "${baseDir}/020.SortMeRNA"

// outdir3= "${baseDir}/030.FastQC"

// outdir4= "${baseDir}/040.STARalign"

// outdir5= "${baseDir}/050.Stringtie"

// outdir6= "${baseDir}/060.Trinity"

// outdir7= "${baseDir}/070.rnaSPAdes-fr"

// outdir8= "${baseDir}/080.PASA"

// outdir9= "${baseDir}/090.VCF"

//

// // Email for notifications

// email='youremail@email.com'

// }

//

// // Options to use with job scheduler and specify memory and retry limits for

// // specific steps of the pipeline.

// // Note that the LSF executor also submits to OpenLava.

// process{

// executor= 'lsf'

// maxRetries= 3

// errorStrategy= 'retry'

// withLabel: Align {

// memory=30.GB

// maxRetries= 3

// }

// withLabel: rnaSPAdes {

// memory= { 50.GB * task.attempt }

// maxRetries= 2

// }

// withLabel: Trinity {

// memory= { 200.GB * task.attempt }

// maxRetries= 2

// }

//

// }

// }

//

//

// sample { // To process a sample (subset) of the input

// params.samp=true

// params.size=5000 // No. of reads to sample

// }

//

//

// stopQCfail { // Avoids exit error due to QC fail

// params.ignoreQC=true

// }

//

//

// skipClean { // Skips the trimmomatic, sortmerna and FastQC steps,

// // thus effectively beginning from alignment and assembly steps.

// // If using already cleaned files, note that the groups file

// // (groupinfo.csv) should have the file names upto _R1.fastq.gz

// // i.e. including _trimmed_clean

// params.skipClean=true

// }

//

//}

//

//-------------------------------------------------------------------------

// GET INPUT --------------------------------------------------------------

// Set reference files. Error if missing.

genome_file = file(params.genome)

annotation_file = file(params.annot)

group_file = file(params.groupfile)

Channel.from(group_file).splitCsv(skip: 1).into {groups1; groups2; groups3}

if( !annotation_file.exists() ) exit 1, "Missing reference annotation file: ${params.annot}"

if( !genome_file.exists() ) exit 1, "Missing reference genome: ${params.genome}"

if( !group_file.exists() ) exit 1, "Missing file of pairID-group correspondence: ${params.groupfile}"

// Set input channel with either full dataset or sample. Error if empty or missing group info.

if(params.samp==true){

Channel.fromFilePairs(params.reads, flat:true)

.ifEmpty { error "Cannot find any reads matching: ${params.reads}" }

.first()

.splitFastq(by:params.size, limit:params.size, pe:true, compress:true, file:true)

.into{FastqIn; FastqIn1}

if( FastqIn1.join(groups1).count().val != 1 ) {

exit 1,

"""Group info missing in ${params.groupfile}.

Note that the first line of ${params.groupfile} should be a header."""

}

}

else if(params.skipClean==true){

Channel.fromFilePairs(params.cleanreads, flat:true)

.ifEmpty { error "Cannot find any reads matching: ${params.cleanreads}" }

.into{PreviouslyCleaned; FastqIn1; FastqIn}

if( FastqIn1.join(groups1).count().val != groups2.count().val ) {

exit 1,

"""At least one set of reads is missing group info in ${params.groupfile}.

Note that the first line of ${params.groupfile} should be a header."""

}

}

else {

Channel.fromFilePairs(params.reads, flat:true)

.ifEmpty { error "Cannot find any reads matching: ${params.reads}" }

.into{FastqIn; FastqIn1}

if( FastqIn1.join(groups1).count().val != groups2.count().val ) {

exit 1,

"""At least one set of reads is missing group info in ${params.groupfile}.

Note that the first line of ${params.groupfile} should be a header."""

}

}

// CLEAN INPUT ----------------------------------------------------------

// Quality trimming and adapter removal with Trimmomatic

process Trimmomatic {

// echo true

module "java:Trimmomatic/0.36"

publishDir params.outdir1, mode: 'copy'

cache 'deep'

input:

set pair_id, file(fqread1), file(fqread2) from FastqIn

output:

set pair_id, file('*_trimmed*R1*'), file('*_trimmed*R2*') into Trimmed

// This outputs paired reads only

when:

params.skipClean!=true

script:

"""

java -jar /software/bioinformatics/Trimmomatic-0.36/trimmomatic.jar \

PE \

${fqread1} \

${fqread2} \

${pair_id}_trimmed_R1.fastq.gz \

${pair_id}_discard_R1.fastq.gz \

${pair_id}_trimmed_R2.fastq.gz \

${pair_id}_discard_R2.fastq.gz \

ILLUMINACLIP:${params.Adapterfile}${params.Pclip} \

LEADING${params.Leading} \

TRAILING${params.Trailing} \

SLIDINGWINDOW${params.SlidWindow} \

MINLEN${params.MinL}

rm ${pair_id}_discard_R1.fastq.gz ${pair_id}_discard_R2.fastq.gz

"""

}

// rRNA removal with SortMeRNA

process SortMeRNA {

//echo true

module "sortmerna/2.1"

publishDir params.outdir2, mode: 'copy'

cache 'deep'

input:

set pair_id, file(trread1), file(trread2) from Trimmed

output:

set pair_id, file('*_clean_R1*'), file('*_clean_R2*') into TrimmedAndSorted

file('*_rRNA*')

when:

params.skipClean!=true

script:

"""

zcat ${trread1} > ${pair_id}_R1.fq

zcat ${trread2} > ${pair_id}_R2.fq

merge-paired-reads.sh \

${pair_id}_R1.fq \

${pair_id}_R2.fq \

${pair_id}_merged.fastq

sortmerna -a 1 --log \

--ref ${params.SORTMERNADB} \

--reads ${pair_id}_merged.fastq \

--paired_in --fastx --otu_map \

--aligned ${pair_id}_rRNA \

--other ${pair_id}_clean

awk 'length(\$0)>0' ${pair_id}_clean.fastq \

> ${pair_id}_clean_chk.fastq

unmerge-paired-reads.sh \

${pair_id}_clean_chk.fastq \

${pair_id}_trimmed_clean_R1.fastq \

${pair_id}_trimmed_clean_R2.fastq

gzip ${pair_id}_trimmed_clean_R1.fastq

gzip ${pair_id}_trimmed_clean_R2.fastq

rm ${pair_id}_R1.fq ${pair_id}_R2.fq \

${pair_id}_clean.fastq ${pair_id}_clean_chk.fastq \

${pair_id}_merged.fastq ${pair_id}_rRNA.fastq

"""

}

// Multiply the clean reads channel to feed into various processes

if(params.skipClean==true){

PreviouslyCleaned.into {Clean1; Clean2; Clean3}

}

else {

TrimmedAndSorted.into {Clean1; Clean2; Clean3}

}

// Quality check with FastQC

process FastQC {

//echo true

module "FastQC/0.11.7"

publishDir params.outdir3, mode: 'copy'

cache 'deep'

input:

set pair_id, file(c1read1), file(c1read2) from Clean1

output:

file "*_fastqc.{zip,html}" into FastQCresult // QCreports

when:

params.skipClean!=true

script:

"""

fastqc ${c1read1} ${c1read2}

"""

}

// Collate FastQC results with MultiQC

process MultiQC {

//echo true

module "MultiQC/1.3"

publishDir params.outdir3, mode: 'copy'

cache 'deep'

input:

file(fastqc) from FastQCresult.collect()

output:

file "multiqc_data/multiqc_fastqc.txt" into MultiQC_det // FastQC details

file "multiqc_report.html"

when:

params.skipClean!=true

script:

"""

multiqc .

"""

}

// Check for remaining adapter content and over 70% read duplication.

// Exit and return error if reads do not pass this check.

// Pipeline can be run with -profile stopQCfail to avoid this check.

process QCcheck {

echo true

cache 'deep'

input:

file(mqcdetails) from MultiQC_det

output:

stdout QCfailVerdict

when:

params.skipClean!=true

script:

if( params.ignoreQC!=true )

"""

sed "s/\t/,/g" $mqcdetails \

| awk -F "," 'BEGIN{dedup=99; ac=99; call="unknown"} \

{if (NR==1) {for (i=1;i<=NF;i++) \

{if (\$i=="total_deduplicated_percentage") {dedup=i} \

else if (\$i=="adapter_content") {ac=i} } } \

else {if ((0 + \$dedup < 30)||(\$ac!="pass")) {call="Y"} \

else {call="N"} } } \

END{print call}'

"""

else

"""

echo "N"

"""

}

if( QCfailVerdict.splitText().count( ~/Y/ ).val > 0 ) exit 1, "Failed QC"

// GENOME-GUIDED GENE ANNOTATION ------------------------------------------

// Generate STAR index if not already present

process Index {

// echo true

module "STAR/2.6.1a"

publishDir params.outdir4, mode: 'copy'

cache 'deep'

input:

file^13^ from genome_file

file(annot) from annotation_file

output:

file("STARindex*bp") into STARindex // Reference genome index

script:

if( params.star.index==' ')

"""

STAR --runMode genomeGenerate \

--genomeDir STARindex${params.star.ReadLen}bp \

--genomeFastaFiles ${genome} \

--sjdbGTFfile ${annot} \

--limitGenomeGenerateRAM ${params.star.IndexRAM} \

--genomeChrBinNbits ${params.star.IndexBinBits} \

--sjdbOverhang ${params.star.IndexSjdbOver} \

--sjdbGTFtagExonParentTranscript ${params.star.IndexSjdbTrans} \

--sjdbGTFtagExonParentGene ${params.star.IndexSjdbGene}"

"""

else

"""

ln -s ${params.star.index} STARindex${params.star.ReadLen}bp

"""

}

if( !STARindex.exists() ) exit 1, "Missing STAR index: ${params.star.index}"

// Align with STAR Two Pass Mode

process StarAlignment {

module "STAR/2.6.1a"

publishDir params.outdir4, mode: 'copy'

cache 'deep'

input:

set pair_id, file(c2read1), file(c2read2) from Clean2

file(annot) from annotation_file

file(index) from STARindex

output:

set pair_id, file("*.bam") into BAM1 // For Stringtie

file("*.bam") into BAM2 // For SNP calling

set pair_id, file("*_Log.final.out") into STARstats

file("*_Unmapped*")

file("*_ReadsPerGene.out.tab")

"""

STAR --runMode alignReads \

--genomeDir ${index} \

--readFilesCommand zcat \

--readFilesIn \

${c2read1} ${c2read2} \

--sjdbGTFfile ${annot} \

--outSAMtype BAM SortedByCoordinate \

--outMultimapperOrder Random \

--outSAMattrIHstart 0 \

--outSAMstrandField intronMotif \

--outReadsUnmapped Fastx \

--outFileNamePrefix ${pair_id}_ \

--quantMode GeneCounts \

--twopassMode Basic \

--alignSoftClipAtReferenceEnds No \

--chimSegmentMin ${params.star.SegmMin} \

--chimJunctionOverhangMin ${params.star.JunctMin} \

--alignMatesGapMax ${params.star.GapMax} \

--alignIntronMax ${params.star.IntronMax} \

--outQSconversionAdd ${params.star.outQSconvAdd}

"""

}

// Collate key points from the STAR mapping logs

collFile = file("Collated_Log.final.out.txt")

collFile.text = "Pair ID\t\

Number of input reads\t\

Uniquely mapped reads number\t\

Number of reads mapped to multiple loci\t\

Number of reads mapped to too many loci\t\

% of reads unmapped: too many mismatches\t\

% of reads unmapped: too short\t\

% of reads unmapped: other\n"

process StarStats {

echo true

publishDir params.outdir4, mode: 'copy'

cache 'deep'

input:

set pair_id, file(stats) from STARstats

file(coll) from collFile

output:

file(coll)

"""

awk -F "\t" 'BEGIN{OFS="\t";input=0;uniq=0;mult=0;many=0;\

mism="";short="";other=""} \

{if ( index(\$1,"Number of input reads |")!=0 ) {input=\$2} \

else if ( index(\$1,"Uniquely mapped reads number |")!=0 ) {uniq=\$2} \

else if ( index(\$1,"Number of reads mapped to multiple loci |")!=0 ) {mult=\$2} \

else if ( index(\$1,"Number of reads mapped to too many loci |")!=0 ) {many=\$2} \

else if ( index(\$1,"% of reads unmapped: too many mismatches |")!=0 ) {mism=\$2} \

else if ( index(\$1,"% of reads unmapped: too short |")!=0 ) {short=\$2} \

else if ( index(\$1,"% of reads unmapped: other |")!=0 ) {other=\$2} } \

END{ print "${pair_id}", input, uniq, mult, many, mism, short, other }' \

${stats} >> ${coll}

"""

}

// Get genome annotations with Stringtie

process Stringtie {

// echo true

module "stringtie/1.3.3b.Linux_x86_64"

publishDir params.outdir5, mode: 'copy'

cache 'deep'

input:

set pair_id, file(bam) from BAM1

file(annot) from annotation_file

output:

file("*_Aligned.gtf") into StringtieGTF

"""

stringtie \

${bam} \

-G ${annot} \

-o ${pair_id}_Aligned.gtf \

-m 100 \

-A ${pair_id}_geneAbundance.tab

"""

}

// Merge stringtie annotations into final file

process StringtieMerge {

// echo true

module "stringtie/1.3.3b.Linux_x86_64"

publishDir params.outdir5, mode: 'copy'

cache 'deep'

input:

file(gtf) from StringtieGTF.toSortedList()

output:

file("Combined_Aligned.gtf") into StrMergeGTF

"""

stringtie --merge \

-o Combined_Aligned.gtf \

-F 0 -T 0 -f 0 -g 0 -i \

${gtf}

"""

}

// GENOME-BLIND DE NOVO ASSEMBLY ---------------------------------------------

// Use information from groupinfo.csv file to group clean reads channel

Clean3.join(groups3)

.groupTuple(by: [3])

.into {groupedReads1; groupedReads2}

// Assembly with rnaSPAdes

process RnaSPAdes {

module "SPAdes/3.13.0:fastx_toolkit/0.0.13"

publishDir params.outdir7, mode: 'copy'

cache 'deep'

clusterOptions '-R rusage[mem=100000]'

input:

set pair_id, file(g1read1), file(g1read2), group from groupedReads1

output:

file("*/*_rnaSPAdes.fasta") into RnaSPAdesOut

file("*/dataset.info")

file("*/spades.log")

file("*/params.txt")

"""

counter=0

CMD='rnaspades.py '

GROUPFILES=($g1read1)

if [ \${#GROUPFILES[@]} -lt 10 ] < 10; then

for lib in ${g1read1}; do

LIB=`ls \$lib | sed 's/_R1.fastq.gz//'`

counter=\$((counter+1))

CMD="\$CMD `echo '--pe'\$counter'-1 '\$LIB'_R1.fastq.gz --pe'\$counter'-2 '\$LIB'_R2.fastq.gz --pe'\$counter'-fr '`"

done

CMD="\$CMD `echo '--ss-rf -m 100 -o '${group}`"

else

zcat ${g1read1} | awk '\$0!=""' | gzip -vc > ${group}_merged_R1.fastq.gz

zcat ${g1read2} | awk '\$0!=""' | gzip -vc > ${group}_merged_R2.fastq.gz

CMD="\$CMD `echo '-1 '${group}'_merged_R1.fastq.gz -2 '${group}'_merged_R2.fastq.gz --ss-rf -m 100 -o '${group}`"

fi

\$CMD

fasta_formatter -i ${group}/transcripts.fasta |\

sed "s/>/>${group}_/" \

> ${group}/${group}_rnaSPAdes.fasta

"""

}

// Assembly with Trinity

process Trinity{

// module "trinityrnaseq/2.6.5"

publishDir params.outdir6, mode: 'copy'

cache 'deep'

clusterOptions '-R rusage[mem=150000]'

input:

set pair_id, file(g2read1), file(g2read2), group from groupedReads2

output:

file("*_trinity/*_trinity.fasta") into TrinityOut

file("*_trinity/Trinity.timing")

"""

module unload perl

module load trinityrnaseq/2.6.5

LEFTREADS=`echo ${g2read1} | sed 's/ /,/g'`

RIGHTREADS=`echo ${g2read2} | sed 's/ /,/g'`

/software/bioinformatics/trinityrnaseq-2.6.5/Trinity \

--seqType fq \

--left \$LEFTREADS \

--right \$RIGHTREADS \

--SS_lib_type RF \

--max_memory 150G \

--min_contig_length 100 \

--output ${group}_trinity

sed "s/>/>${group}_/" ${group}_trinity/Trinity.fasta \

> ${group}_trinity/${group}_trinity.fasta

"""

}

// ANNOTATION REFINEMENT WITH PASA --------------------------------------------

// Run PASA on combined Trinity and rnaSPAdes input

process PASA{

//module "PASA/2.3.3:BBMap/37.93:samtools:fasta"

publishDir params.outdir8, mode: 'copy'

cache 'deep'

input:

file(stringtie) from StrMergeGTF

file^13^ from genome_file

file(rnaSPAdes) from RnaSPAdesOut.collect()

file(trinity) from TrinityOut.collect()

output:

file("*assemblies.fasta")

file("*pasa_assemblies.gtf")

file("*pasa_assemblies_described.txt")

file("*pasa_alignment_assembly_building.ascii_illustrations.out")

file("*polyAsites.fasta")

"""

module unload perl

module load PASA/2.3.3 BBMap/37.93

echo "DATABASE=${params.PASA.database}\n${params.PASA.options}" \

> alignAssembly.config

cat ${rnaSPAdes} ${trinity} > PASAinput.fasta

dedupe.sh \

in=PASAinput.fasta \

out=PASAinput_dedupe.fasta \

csf=PASAinput_dedupe_clusters.txt \

absorbcontainment=f

rm PASAinput.fasta

seqclean PASAinput_dedupe.fasta

perl -I /software/bioinformatics/PASA-2.3.3/PerlLib \

/software/bioinformatics/PASA-2.3.3/Launch_PASA_pipeline.pl \

-c alignAssembly.config \

-C -R \

-g ${genome} \

-t PASAinput_dedupe.fasta.clean \

-T \

-u PASAinput_dedupe.fasta \

--ALIGNERS gmap \

--MAX_INTRON_LENGTH ${params.PASA.maxIntron} \

--trans_gtf ${stringtie} \

--transcribed_is_aligned_orient

"""

}

// DIVERGENCE ESTIMATION USING SNPS -------------------------------------------

// Call SNPs with mpileup and bcftools

process CallSNPs {

// echo true

module "samtools/1.7:bcftools/1.6:bedtools/2.27.1"

publishDir params.outdir9, mode: 'copy'

cache 'deep'

input:

file(bam) from BAM2.toSortedList()

file^13^ from genome_file

output:

file("SNPcoverage.out")

file("Aligned_merged.vcf.gz")

file("Aligned_merged_filt.vcf")

file("Aligned_merged_genomecov.bedgraph")

script:

"""

samtools merge Aligned_merged.bam ${bam}

samtools mpileup -vgf ${genome} ${bam} \

| bcftools call -vmO z -o Aligned_merged.vcf.gz

bcftools view Aligned_merged.vcf.gz \

| vcfutils.pl varFilter - > Aligned_merged_filt.vcf

echo "Datatset\tSNPs detected\tBases with 0 coverage\tBases with 1 read coverage\tTotal bases in genome\tBases evaluated for SNPs" \

> SNPcoverage.out

awk '/#/{next;} {count++} END{printf "${baseDir}\t"count"\t"}' Aligned_merged_filt.vcf \

>> SNPcoverage.out

bedtools genomecov -split -ibam Aligned_merged.bam \

> Aligned_merged_genomecov.bedgraph

awk -F "\t" '(\$1=="genome"){total+=\$3; \

if (\$2==0) {a=\$3; printf a"\\t"} \

else if (\$2==1) {b=\$3; printf b"\\t"}} \

END{printf total"\\t"total-a-b"\\n"}' \

Aligned_merged_genomecov.bedgraph \

>> SNPcoverage.out

"""

}

// EMAIL NOTIFICATION ---------------------------------------------------------

// Email notification when the workflow is completed or aborted by an error

workflow.onComplete {

def subject = 'My pipeline execution'

def recipient = params.email

['mail', '-s', subject, recipient].execute() << """

Pipeline execution summary

---------------------------

Completed at: ${workflow.complete}

Duration : ${workflow.duration}

Success : ${workflow.success}

workDir : ${workflow.workDir}

exit status : ${workflow.exitStatus}

Error report: ${workflow.errorReport ?: '-'}

"""

}
