# Supplementary material for: Genomic analysis uncovers functional variation in the C-terminus of anthocyanin-activating MYB transcription factors
Source: Hortic Res. 2021 Apr 1;8:77. doi: 10.1038/s41438-021-00514-1 (PMC8012628; doi:10.1038/s41438-021-00514-1)

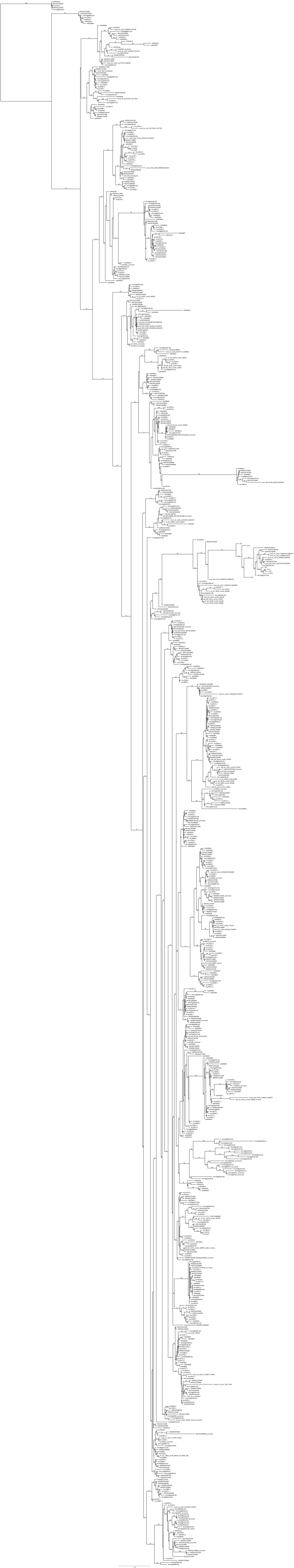

Supplement: Supplementary file 3 — Fig S3 [file 41438_2021_514_MOESM3_ESM.pdf]
